# How Progressive Hydrogenation Shapes the Optical Response of Carbon Clusters

**DOI:** 10.1021/acs.jpca.6c03043

**Published:** 2026-10-07

**Authors:** Francesca D’Ambrosio, Alice Frustaci, Enrico Bodo

**Affiliations:** Chemistry Department, 9311 University of Rome “La Sapienza”, P. Aldo Moro 5, Rome 00185, Italy

## Abstract

Hydrogenated carbon clusters represent structural intermediates conveniently placed between isolated molecular hydrocarbons and extended carbon materials in which hydrogen content and chemical functionalization critically determine both structure and optical response. In this work, we present a computational investigation of medium-sized carbon clusters (ranging from 20 to 35 carbon atoms) with different degrees of hydrogenation, nitrogen doping, and amino-functionalization. A fairly large ensemble of structures was generated using ab initio methods showing that hydrogenation induces a significant structural reorganization, progressively disrupting π-conjugation and increasing sp^3^ character. Time-dependent density functional theory calculations were carried out for the C_30_ family of clusters, showing that increasing hydrogenation induces a systematic blue shift of the absorption spectra and a depletion of low-energy transitions, consistent with the reduction of π-conjugated domains. In contrast, nitrogen doping and amino-functionalization partially counteract this effect by inducing the presence of localized electronic states that produce absorption in the visible range. Partially hydrogenated C30 clusters exhibit large Stokes shifts, further enhanced by amino functionalization, while N-doping promotes visible emission and excited-state localization. We conclude that hydrogenation and nitrogen doping generally act as two complementary driving forces on the optical properties of carbon clusters and disordered carbon-based systems.

## Graphical abstract

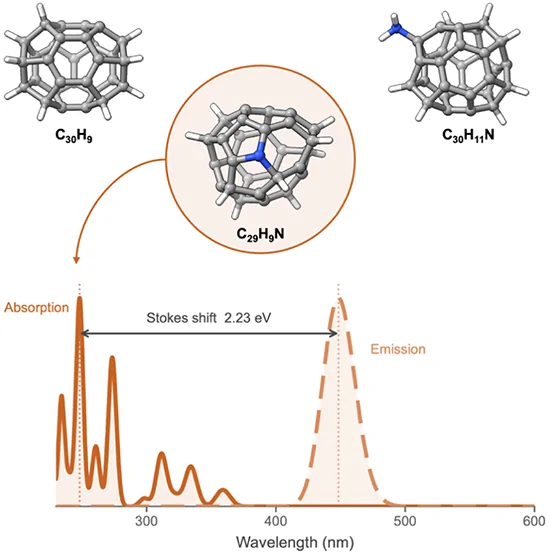

## 1 Introduction

Carbon clusters have gathered a significant amount of attention, because they are conveniently placed between small molecular-sized systems, such as polycyclic aromatic hydrocarbons (PAHs), and bulk carbon materials such as graphene and graphite. Consequently, they are of interest in a multitude of scientific fields, ranging from combustion chemistry and soot formation to hydrogenated amorphous carbon (HAC) substrates, from carbon-based nanomaterials to astrochemistry.

In combustion, soot formation proceeds through the growth and aggregation of polycyclic aromatic hydrocarbons (PAHs) and related carbonaceous intermediates. The hydrogenation degree of these species strongly influences their reactivity, curvature, and tendency to form larger aggregates. Therefore, partially hydrogenated clusters can be regarded as transient species whose hydrogen content determines their evolution toward amorphous graphitic systems or their fragmentation into smaller nanostructures.^1–5^

In astrophysics, hydrogenation and dehydrogenation processes are fundamental in understanding the molecular evolution of carbonaceous species.^6–8^ Observations support a scenario in which progressive hydrogen loss from small aromatic molecular species leads to closed fullerene cages under UV irradiation.^9^ Partially or fully saturated carbon structures are components of hydrogenated amorphous carbon (HAC), an ingredient of interstellar dust.^10,11^

In the context of carbon-based materials technology, hydrogenation is a key strategy for tuning the electronic structure and stability of carbon nanostructures,^12^ including ionization potentials, open-shell character, and the π-manifold in finite clusters.^13^

Hydrogenation can have a dramatic role also in the chemistry of carbon nanodots (CNDs), a particular class of carbon-based nanoparticles. Sciortino et al.^14^ demonstrated how surface passivation fundamentally alters the electronic structure of CNDs and reshape their electronic structure by creating new surface-localized emissive states that govern their photophysical behavior.

Some caution is due when comparing the present findings with the extensive experimental literature on carbon nanoparticles, particularly CNDs. Such a comparison will be made below, but an important caveat must first be considered when relating the present models to experimental systems. CNDs photoluminescence may include contributions from molecular fluorophores formed during synthesis and retained after incomplete purification.^15,16^ Molecular and core-related emission channels coexist and their relative importance depends on the synthetic conditions.^17,18^ The present clusters represent exclusively the conjugated carbonaceous domains and therefore probe their intrinsic optical response, without accounting for additional molecular fluorophores.

The studies discussed above motivate further investigation of hydrogenated carbon clusters (HCC), whose structural diversity arises from the interplay of π-conjugation, curvature, and local hybridization. In our previous works,^19,20^ this interplay was extensively investigated considering pure carbon clusters, doped and functionalized. The next logical step is to consider the role of hydrogenation in altering the conformation and electronic structure of carbon clusters. From basic organic chemistry, it is obvious that the expected effect of introducing hydrogen is to induce sp^2^ → sp^3^ change of hybridization of the carbon atoms, and thus modify electronic gaps, geometry and optical properties. While this is rather well-known for extended carbon systems, in finite clusters, the effect of hydrogenation can be even more dramatic, causing a complete change of the potential energy surface due to bond saturation and conjugation disruption, ultimately altering the geometry, making cage-like isomers become planar, for example.^13^

From the theoretical point of view, various studies were conducted on HCCs, but they typically focused on small systems. As already pointed out, C_n_H_m_ species show a large structural diversity within a limited energy range, and the hydrogen presence induces a spin multiplicity change with respect to the parent unsaturated species. Bennedjai et al.^21^ with high-level *ab initio* investigation of C_3_H, C_5_H and their respective ions proved that even such small systems show a multitude of topologies where their stability is determined by the positions of the hydrogen atom. On a slightly larger scale, extensive studies of the C_24_H_n_ family, conducted by Pla et al.,^13^ have shown that hydrogen has an active role in determining the structure, from planar “flake-like” motifs, to three-dimensional “fullerene-like” arrangements. Robertson et al.^22^ showed that the optical band gap of amorphous carbon is governed by the size of π-conjugated domains and it was shown that emission originates from localized recombination processes within sp^2^ regions.^23^

The topology of the clusters’ structures also affects their optical properties, as shown by experimental and theoretical studies.^24,25^ In particular, linear and monocyclic carbon clusters show a different optical response, with linear chains having a strong absorption in UV–vis and NIR regions, with increasing intensity as their chain length increases. These findings are further supported by large-scale simulations of carbon cluster isomers, which demonstrate that sp^2^-rich structures dominate the optical response in the UV–visible region through π → π* transitions, while variations in morphology and disorder give rise to distinct spectral fingerprints.^26–28^

In our previous work,^20^ we investigated the absorption properties of unsaturated carbon clusters, both pristine and doped with graphitic nitrogen. Here, we build on these models, and we examine the effect of hydrogenation also on their absorption and emission properties. In this work, we present an investigation of the structural and energetic evolution of medium-sized carbon clusters (C_20_–C_35_) under progressively increasing hydrogenation. For each size and hydrogenation ratio, multiple isomers were generated, enabling a statistical characterization of the accessible structural ensemble. For a specific family, C_30_H_n_, we evaluated the optical properties, both for absorption and emission phenomena, considering the effect of doping (graphitic nitrogen), functionalization (−NH_2_) and changing H/C ratio (from 0 to 0.5).

## 2 Methods

### 2.1 Structure Generation

Partially hydrogenated carbon clusters were generated starting from the set of unsaturated carbon structures reported in our previous work.^19^ The original systems included pristine (C_n_), nitrogen-doped (C_n–1_N), and NH_2_-functionalized (C_n_NH_2_) clusters, with sizes ranging from n = 20 to n = 35 carbon atoms.

Here we explore different degrees of hydrogenation of those structures by progressively increasing the H/C ratio, simply defined as the ratio between the total number of hydrogens and carbon atoms. The range of the H/C ratio went from *medium* saturation (H/C = 0.25–0.31), *high* saturation (H/C = 0.35–0.37), and finally to the *maximum* degree considered in this study (H/C = 0.48–0.55). The maximum value of the H/C ratio was not further pushed to higher values (2 of alkenes, for example) because we needed to preserve the three-dimensional cage-like structures, and with high H/C ratio the dominant structure is a linear chain.

All hydrogenated structures underwent a conformational search using the GOAT-EXPLORE^29^ algorithm in ORCA^30^ combined with xTB semiempirical energy,^31,32^ similarly to how it was done for the original starting pristine structures. This procedure is convenient because it allows for bond position changes and leads not only to a set of conformers, but isomers as well. Each calculation provided an ensemble of structures sorted by energy from which five representative isomers were selected using a structural diversity criterion rather than an energy-based one. Specifically, a farthest-point sampling (max–min) procedure based on RMSD was applied after optimal alignment via the Kabsch algorithm.^33^ In each ensemble, the lowest-energy structure was chosen as the initial seed, and additional isomers were iteratively selected by maximizing their RMSD with respect to the already selected set. This strategy ensures that the five chosen configurations are maximally dissimilar and span a broad portion of the accessible structural space. The resulting pairwise RMSD values among the selected isomers turned out to be between 1.0 and 3.7 Å.

In total, around 4000 structures were obtained from the isomeric search. These structures were organized in batches for three families: C_n_H_m_ (pristine), C_n–1_H_m_N (N-doped), and C_n_H_m+2_N (or C_n_H_m_-NH_2_, functionalized) each grouped into 4 sizes with n = 20, 25, 30, 35, and 3 saturation degrees. Five isomers from each family, dimension and saturation were then selected using the method above for a total of 180 candidates, as summarized in Figure 1. These structures were optimized at the DFT level using the ωB97X-D3 functional in combination with the def2-TZVP basis set.^34,35^ Coulomb and exchange contributions were treated using the RIJCOSX approximation in conjunction with the def2/J auxiliary basis set.^36,37^ At this point, a structural analysis was conducted on all isomers, as reported in Section 3.

**Figure 1:**
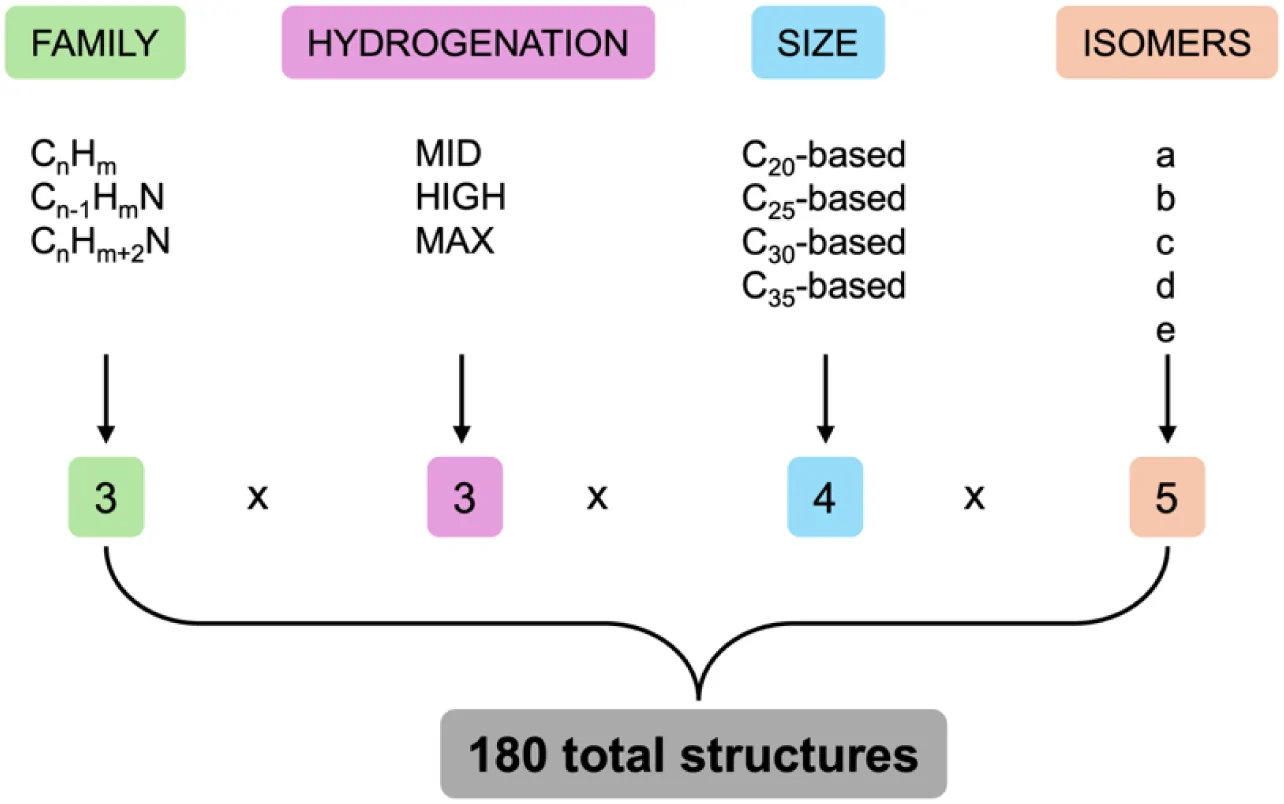
Schematic overview of the data set construction for partially hydrogenated carbon clusters. Structures are generated by combining different families (pristine, N-doped, and NH_2_-functionalized), three degrees of hydrogenation (MID, HIGH, MAX), four size classes (C_20_, C_25_, C_30_, and C_35_), and five representative isomers selected for each case. The final data set is obtained as the product of these independent variables, resulting in a total of 180 structures.

### 2.2 Absorption Calculations

The optimized DFT geometries for the C_30_ subset, covering all doping/functionalization variants and hydrogenation degrees considered, were further selected as the exemplar cases to calculate absorption and emission spectra. Excited-state properties were evaluated at the ωB97X-D3/def2-TZVP level using TD-DFT. The choice of the method is justified by sensitivity test ran for our previous work on similar clusters.^20^ All TD-DFT calculations were performed within the Tamm–Dancoff approximation (TDA), as per ORCA’s default settings.^38^

For the C_n_H_m_ clusters the ground state is a doublet spin system (S = 1/2), therefore excited states calculations were carried out within the unrestricted formalism, yielding spin-conserving doublet excitations. In contrast, nitrogen-doped and NH_2_-functionalized clusters exhibit a singlet ground state (S = 0), and only singlet–singlet transitions were considered using the restricted framework. The different spin states arise from the hydrogenation protocol adopted. In other words, we privileged an equal degree of saturation (among neat and nitrogen doped clusters) over a uniform final spin multiplicity. For UKS calculations, the expectation value of the total spin operator, ⟨S^2^⟩, for the open-shell doublet clusters was monitored. Most structures show only modest deviations from the ideal value, with only a subset of isomers exhibiting appreciable spin contamination.

The choice of considering only singlets and doublets is motivated by our previous study conducted on pure C_n_ clusters, in which the singlet and triplet spin configurations were found to be very close in energy (for sufficiently large n) and exhibited essentially identical structures.^19^ Also, the triplet-singlet energy difference turned out to be few tens of meV per carbon atom. This energy is much smaller than the variation in atomization energy per atom induced by hydrogenation, indicating that the main energetic trend is primarily controlled by the degree of hydrogenation rather than by spin effects.

Solvent effects were included in all the TDDFT calculations using the CPCM model with the standard parameters for water.^39^ All spectra were convoluted using the same Gaussian broadening, applying a constant full width at half-maximum (fwhm) of 0.12 eV to each transition to enable consistent comparison of spectral shapes and intensities.

### 2.3 Emission Calculations

Calculation of emission spectra has been performed using the same methodological setup that we described above for absorption. To establish the computational protocol for the emission calculations, a preliminary excited-state exploration was performed on representative C_20_-based models belonging to the three chemical classes considered in this work (pristine, N-doped, and NH_2_-functionalized) with an H/C ratio of 0.25. For each model, the first 25 vertical excitation energies were initially computed with TD-DFT at the ground-state optimized geometry. Candidate excited states for TD-DFT geometry optimizations were selected either as the lowest-energy optically active state or as the states with the highest oscillator strength. Geometry optimizations for these selected excited states were then performed using the TD-DFT gradient. Root following^40,41^ protocol was adopted during excited-state optimizations to ensure that the correct emitting state was consistently tracked along its quasi-diabatic PES. Excited-state emission was evaluated using the vertical energies at the excited state optimized geometries. This preliminary analysis showed that, despite different initially bright states, after a successful optimization, a good candidate for the emissive state (as per the calculated oscillator strength) was consistently associated with the lowest excited states of the same spin multiplicity in accordance with Kasha’s rule.^42^ The results for this test are reported in Tables S1 and S2 of the Supporting Information.

Based on these results, emission spectra for the larger C30-based systems were computed by optimizing selected low-lying excited states of the appropriate multiplicity. For the pristine and NH_2_-functionalized systems, optimization of the lowest excited states (D_1_ and S_1_, respectively) led to emission transitions with negligible oscillator strengths. We therefore also explored optimization starting from D_2_ for the pristine system and S_2_ for the NH_2_-functionalized system. In the pristine system, the state initially identified as D_2_ evolved into D_1_ during the optimization, and emission was consequently evaluated from the resulting D_1_ state. In contrast, the S_2_ state of the NH_2_-functionalized system retained its state identity throughout the optimization and, owing to its appreciable oscillator strength, was considered a viable candidate for emission. A summary of the relevant excited states for these systems is provided in Table S3.

Characterization of the electronic transition density in absorption and emission has been obtained using TheoDORE program.^43^ A Gaussian full width at half-maximum (fwhm) of 0.18 eV was used for the emission bands to qualitatively reflect the broader spectral features expected for emission after excited-state relaxation. The Stokes shift was taken as the difference between the maximum of the broadened absorption spectrum and the maximum of the corresponding broadened emission band.

## 3 Results and Discussion

The progressively hydrogenated clusters were manually generated with a precise number of hydrogen and carbon atoms in mind, to obtain the correct H/C ratios for each composition. To consider this ratio as a continuous parameter, we chose to focus on three main degrees of hydrogenation (called MID, HIGH and MAX from now on). The MID category consists of carbon clusters with an H/C ratio between 0.25 and 0.30, the HIGH category includes clusters with values between 0.35 and 0.37, and the MAX category those with values between 0.48 and 0.55. In total, 36 partially hydrogenated clusters were generated and used as initial input structures for the subsequent conformational search from which a total of 180 structures were then produced. A detailed summary of their composition is reported in Table 1. For each composition, five structural isomers were identified and analyzed.

**Table 1:** Molecular Weights (MW) and Hydrogenation Levels (H/C) and Corresponding Compositions of the Pristine (C_n_H_m_), Nitrogen-Doped (C_n–1_H_m_N), and NH_2_-Functionalized (C_n_H_m+2_N) Carbon Clusters Investigated in this Work^a^

Framework	C_n_H_m_	MW	C_n–1_H_m_N	MW	C_n_H_m+2_N	MW	H/C
**MID** (0.25–0.31)							
C_20_-based	C_20_H_5_	245.3	C_19_H_5_N	247.3	C_20_H_7_N	261.3	0.25
C_25_-based	C_25_H_7_	307.3	C_24_H_7_N	309.3	C_25_H_9_N	323.4	0.28
C_30_-based	C_30_H_9_	369.4	C_29_H_9_N	371.4	C_30_H_11_N	385.4	0.30
C_35_-based	C_35_H_11_	431.5	C_34_H_11_N	433.5	C_35_H_13_N	447.5	0.31
**HIGH** (0.35–0.37)							
C_20_-based	C_20_H_7_	247.3	C_19_H_7_N	249.3	C_20_H_9_N	263.3	0.35
C_25_-based	C_25_H_9_	309.4	C_24_H_9_N	311.3	C_25_H_11_N	325.4	0.36
C_30_-based	C_30_H_11_	371.4	C_29_H_11_N	373.4	C_30_H_13_N	387.4	0.37
C_35_-based	C_35_H_13_	433.5	C_34_H_13_N	435.5	C_35_H_15_N	449.5	0.37
**MAX** (0.48–0.55)							
C_20_-based	C_20_H_11_	251.3	C_19_H_11_N	253.3	C_20_H_13_N	267.3	0.55
C_25_-based	C_25_H_13_	313.4	C_24_H_13_N	315.4	C_25_H_15_N	329.4	0.52
C_30_-based	C_30_H_15_	375.5	C_29_H_15_N	377.5	C_30_H_17_N	391.5	0.50
C_35_-based	C_35_H_17_	437.5	C_34_H_17_N	439.5	C_35_H_19_N	453.5	0.48

a Clusters are classified into MID, HIGH, and MAX hydrogenation regimes. For the amino-functionalized clusters, the two hydrogen atoms of the −NH_2_ group were not considered as passivating atoms, so the ratios are calculated only on the remaining (n – 2) hydrogen atoms of the total composition.

For the sake of visual representation, we report in Figure 2 representative structures for the n = 20 pristine clusters with an increasing level of hydrogen content, specifically three isomers for each hydrogenation level considered (from left to right, MID, HIGH and MAX). As shown by the n = 20 example in Figure 2, the structure of this cluster is open, flake-like and this topology is largely preserved during H atom addition for different isomers at different energies. Despite keeping the overall topology, the increase of the H/C ratio has a significant effect on the ring arrangement and the local topology of the carbon network. A preliminary analysis of the structures other than n = 20, reveals that, just from observing the global shape of the clusters, increasing hydrogenation induces a significant structural reorganization in the bond topology but these changes preserve either the flake-like or cage-like topology of the unsaturated parent cluster.

**Figure 2:**
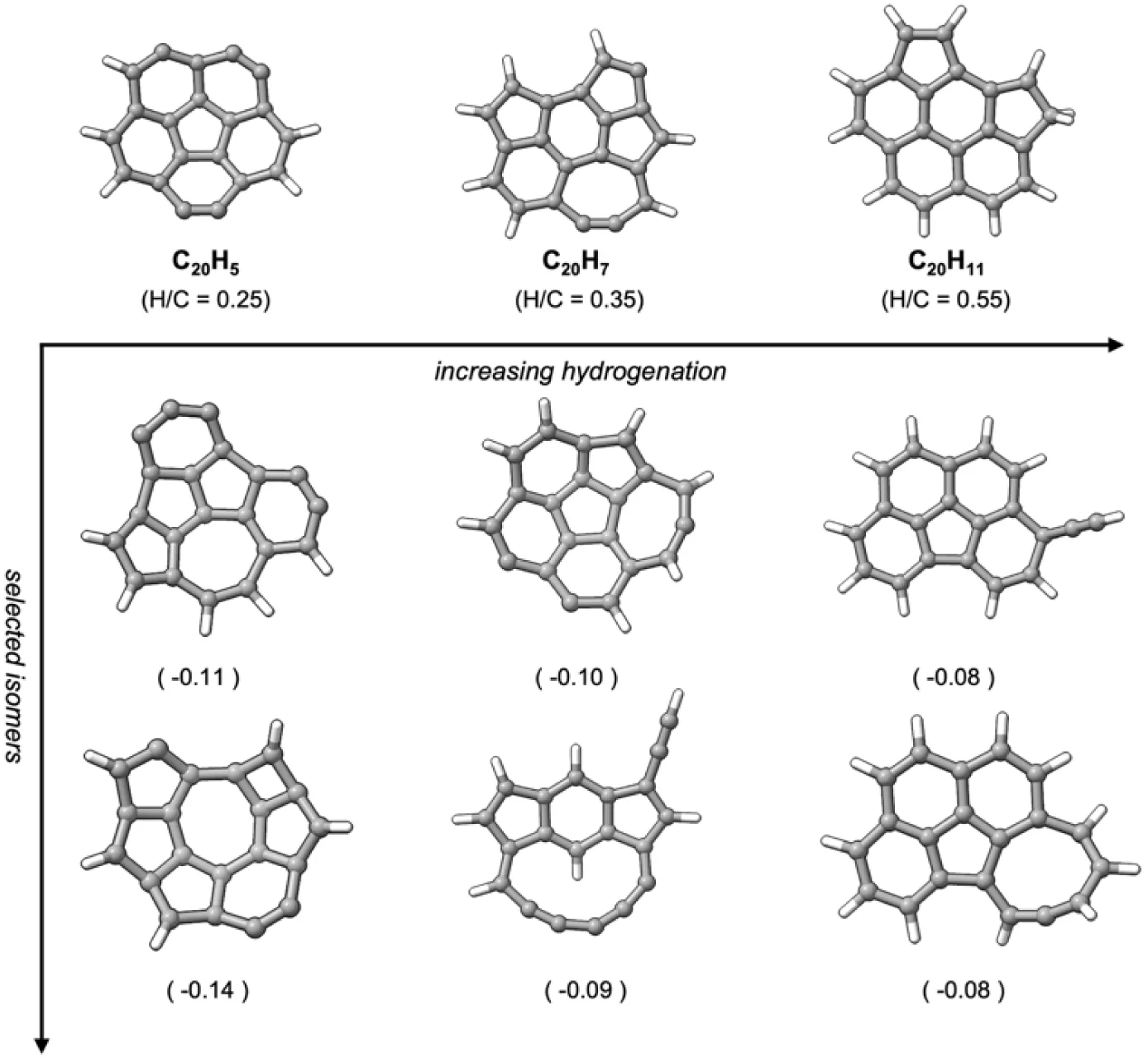
Representative structures illustrating the effect of hydrogenation for pristine C_20_ based clusters. DFT optimized geometries are shown for increasing H/C ratios corresponding to MID, HIGH, and MAX hydrogenation regimes. For each structure shown in the upper part of the figure, selected isomers of the same composition are also reported. Values in brackets indicate the relative atomization energy per atom (in eV/atom), computed with respect to the top structures, which represent the most stable configurations.

We also point out that the molecular weights of the clusters considered here span 245–454 g mol^–1^, i.e., roughly 0.7–1.0 nm of geometric extension (see Table 1). This places them one to two orders of magnitude below typical CNDs, whose cores of 2–10 nm correspond to about 10^3^–10^5^ g mol^–1^. Our systems therefore model the smallest conjugated domains that can be embedded in a carbon-dot core rather than the whole nanoparticle, which is precisely the regime in which the size of the sp^2^ domain, controls the optical gap.

### 3.1 Stability and Structural Analysis

We first examine the dependence of the atomization energy per atom on hydrogen content, that we report in Figure 3. Atomization energy is defined as the total electronic energy of the cluster with respect to its complete fragmentation into its atomic constituents. To obtain a size independent quantity we divide the resulting energy by the number of atoms. Conventionally, it is calculated to be a positive quantity for stable clusters and can be used to trace the energetic stability across families of clusters of different sizes. The values reported in Figure 3 are averages computed over the five selected isomers for each composition. Stability increases with cluster size (across the four panels), as expected, but it decreases with increasing H/C ratio within a given size (within the same panel), indicating that partial hydrogenation has a destabilizing effect across all cluster sizes considered. This behavior is consistent with the fact that graphene resists hydrogenations and that hydrogenation of stable fullerenes is a highly endothermic process. It can be rationalized by pointing out that hydrogenation breaks the extended π-conjugation of the carbon framework, converting sp^2^ carbon atoms into tetrahedral sp^3^ geometries. Consequently, the loss of electron delocalization outweighs the energetic gain associated with dangling bond saturation, resulting in overall less stable systems.

**Figure 3:**
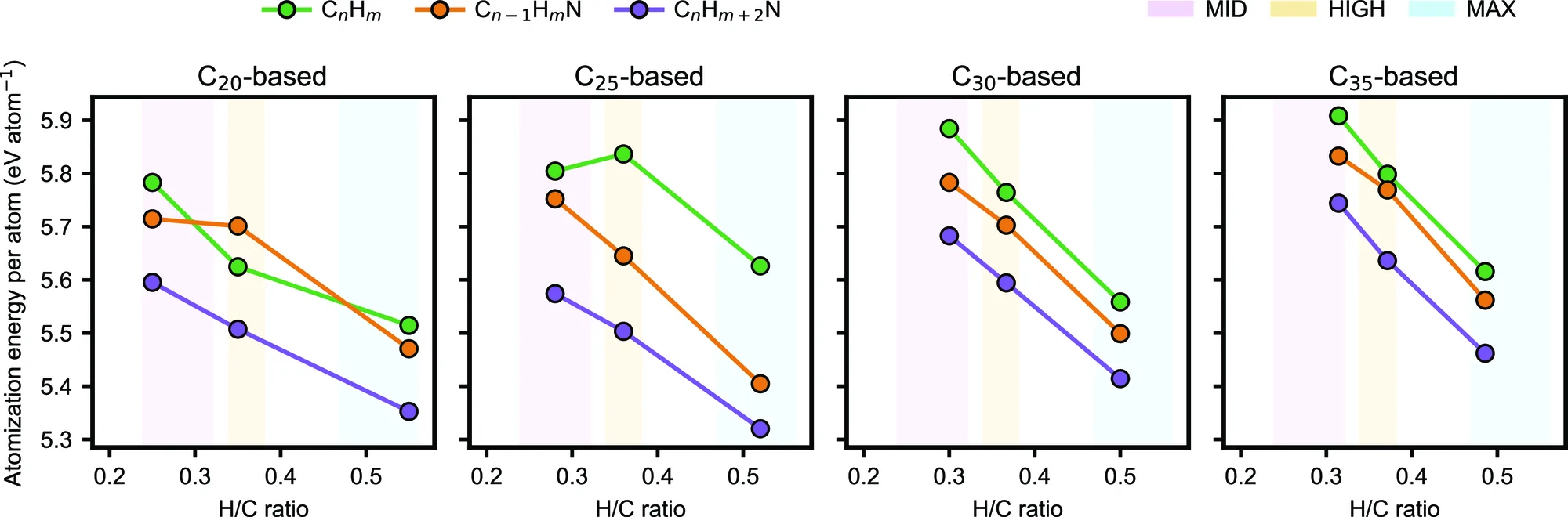
Atomization energy per atom as a function of the H/C ratio for hydrogenated carbon clusters of different nominal sizes. Each panel corresponds to a cluster family based on the carbon framework size (C_20_, C_25_, C_30_ and C_35_ based structures). Each point is the average atomization energy per atom obtained from the five selected isomers for each composition, while lines are only guides connecting structures within the same chemical class. Colors indicate the cluster type: C_n_H_m_ in green, C_n–1_H_m_N in orange and C_n_H_m+2_N in purple. Shaded regions highlight the hydrogenation regimes discussed in the text (MID, HIGH, and MAX).

The second factor to consider is the effect of chemical composition on structural stability, particularly the impact of graphitic nitrogen doping and surface functionalization with an amino group. In almost all cases, both the doped and functionalized systems exhibit reduced stability compared to their pristine counterparts, in contrast to the trend observed for unsaturated clusters, previously investigated.^19^ The two forms of nitrogen incorporation also affect cluster stability to different extents, with the introduction of an −NH_2_ group producing a more pronounced decrease in atomization energy. This can be attributed to the breakage of the conjugated network by the graphitic nitrogen atom, which introduces more localized electronic states and thereby weakens the overall bonding pattern, but also to the intrinsic weakness of the C–NH_2_ bond structure (at least with respect to the C–C and C–H network) that affects the overall atomization energy. Interestingly, the destabilizing effect of hydrogenation appears to be slightly more pronounced for larger clusters (C_30_-based and C_35_-based structures), as we can observe from the slope of the lines connecting the three clusters shown for each class. In these clusters, the initial stabilization provided by extended π-delocalization is greater^19^ and altering this extended conjugation leads to a more significant “drop” in energy.

Finally, the shaded regions (MID, HIGH, MAX) highlight how it is not simple to establish a gradual trend in the decrease in atomization energy when going from the MID to the HIGH or from HIGH to MAX hydrogenation regime with changes in slope appearing nonsystematically. The primary cause for the decrease in energy arises from the initial disruption of the conjugated carbon network, whereas subsequent hydrogenation mainly affects the energy in a nonsimple way due to the induced changes in coordination patterns of the carbon atoms. This fact is supported by the complete energy trend reported in Figure S1 of the Supporting Information, that also includes the unsaturated clusters. Evidently, in Figure S1, the largest gap is observed in the jump from H/C = 0 (unsaturated regime) to the MID regime.

To capture the structural evolution and topological reorganization of these systems, an effective approach was needed to simultaneously represent all the relevant variables considered—size, H/C ratio, topology and nitrogen doping/functionalization. To this end, we employed a PCA analysis along the lines of the one reported by Pla et al.^13^ The structural descriptors are (i) the gyration radius, r_g_, which accounts for the spatial extension of the clusters; (ii) R_5/6_, that is the total number of 5- and 6-membered rings, used as a measure of structural stability and graphitic ordering; (iii) the a-sphericity, β, which captures the distortion induced by the presence of hydrogen and (iv) the extension of the conjugated domain $N_{\mathrm{conj}}^{\max}$ . A more detailed description of these parameters is provided in the Supporting Information.

Figure 4 shows the PCA labeling for all clusters considered, while class-resolved PCAs, along with a detailed analysis of the selected descriptors and their loadings, are reported in Figure S2. The first two principal components (PC1 and PC2) account for 51.1% and 33.7% of the total variance, respectively. PC1 is mainly associated with *R*_5/6_ and *β*, whereas $N_{\mathrm{conj}}^{\max}$ contributes most strongly to PC2 together with *r*_*g*_. Thus, the conjugated-domain size introduces a structural contribution distinct from the purely geometric descriptors.

**Figure 4:**
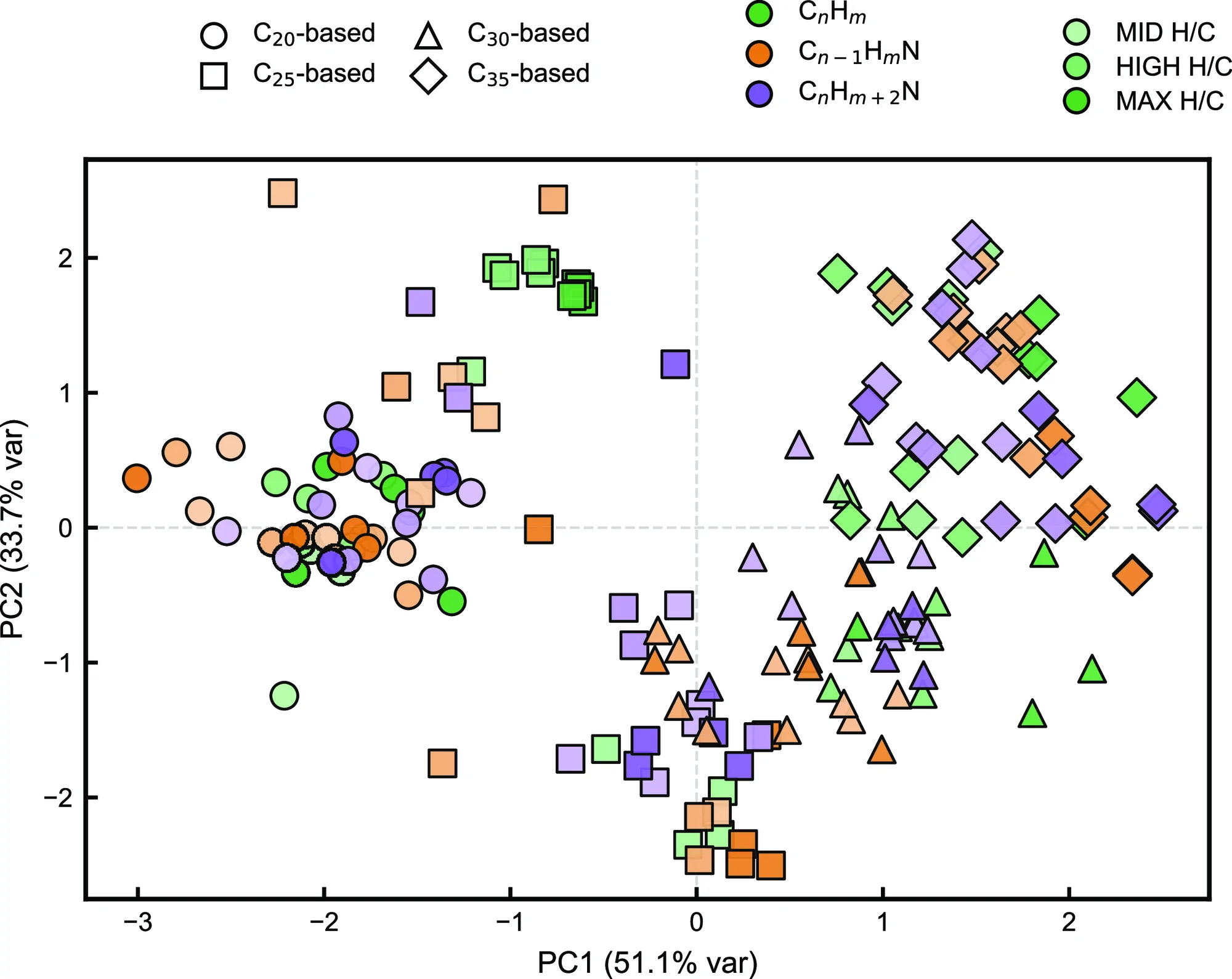
Principal component analysis of the hydrogenated cluster set using four structural descriptors: gyration radius (*r*_*g*_), asphericity parameter (*β*), fused-ring descriptor (*R*_5/6_), and largest conjugated-domain size $\left(N_{\mathrm{conj}}^{\max}\right)$ . Marker shape identifies the cluster size (C_20_-, C_25_-, C_30_-, and C_35_-based), marker color identifies the chemical family (pristine C_n_H_m_, graphitic-N C_n–1_H_m_N, and NH_2_-functionalized C_n_H_m+2_N), and color shade indicates the hydrogenation regime (MID, HIGH, and MAX H/C). PC1 and PC2 account for 51.1% and 33.7% of the total variance, respectively.

Smaller clusters, such as the C_20_-based and C_25_-based structures, are predominantly located at negative PC1 values, while larger systems (C_30_-based and C_35_-based structures) shift toward positive PC1 values. This indicates that PC1 is strongly correlated with cluster size and overall structural complexity, likely reflecting systematic changes in R_5/6_ and asphericity as the clusters become larger. Superimposed on this size effect, hydrogenation also contributes to the redistribution along PC1, although no simple shift with increasing H/C is observed across all chemical families. Structures with higher H/C ratios (MAX regime, darkest color for each category) tend to shift toward more positive PC1 values, suggesting that hydrogenation introduces systematic structural changes that are captured by the principal components, such as increased sp^3^ character and reduced planarity. The second principal component (PC2, 33.7% variance) captures more subtle variations within each size class and is mainly associated with the extension of the largest conjugated domain, $N_{\mathrm{conj}}^{\max}$ , together with the spatial extension of the clusters. The overlap between different compositions within the same size range indicates that composition alone is not sufficient to fully discriminate the structures, and that hydrogenation and geometric arrangement play a comparable role in defining the structural space. The evolution of $N_{\mathrm{conj}}^{\max}$ with H/C is not strictly universal across all cluster sizes, so we have avoided describing the first hydrogenation step as a universal abrupt collapse of conjugation. The dependence of $N_{\mathrm{conj}}^{\max}$ on hydrogenation is size- and topology-dependent: the smaller clusters show a more irregular evolution, whereas a clearer reduction of the largest conjugated domain with increasing H/C is observed for the larger C30- and C35-based systems, particularly in the N-containing families (the relevant data are in Figure S2).

The behavior depicted by this analysis is consistent with previous findings from ref 13, where hydrogen coverage was shown to drive the transition from planar, conjugated carbon frameworks to more distorted and three-dimensional structures. However, compared to that work, our case reveals an additional level of organization associated with heteroatom incorporation and with the balance between ring topology, global shape and conjugation, as we just described.

### 3.2 Absorption and Emission Spectra

To illustrate the optical properties, we computed absorption spectra and emission spectra selecting only a few of the clusters previously analyzed. We focused only on C_30_-based structures for each class, as representative of all medium sized clusters.

Figure 5 shows, for each cluster considered, only the global minimum geometry as a representative structure, whereas the spectra were computed for five selected isomers. The absorption spectra were then averaged over these five isomers for each class, and the resulting averaged spectra are reported in Figure 6. They reveal a consistent evolution of the optical response as a function of both hydrogenation degree and chemical composition. The unique (nonaveraged) absorption spectra for each isomer are reported in the SI in Figure S3.

**Figure 5:**
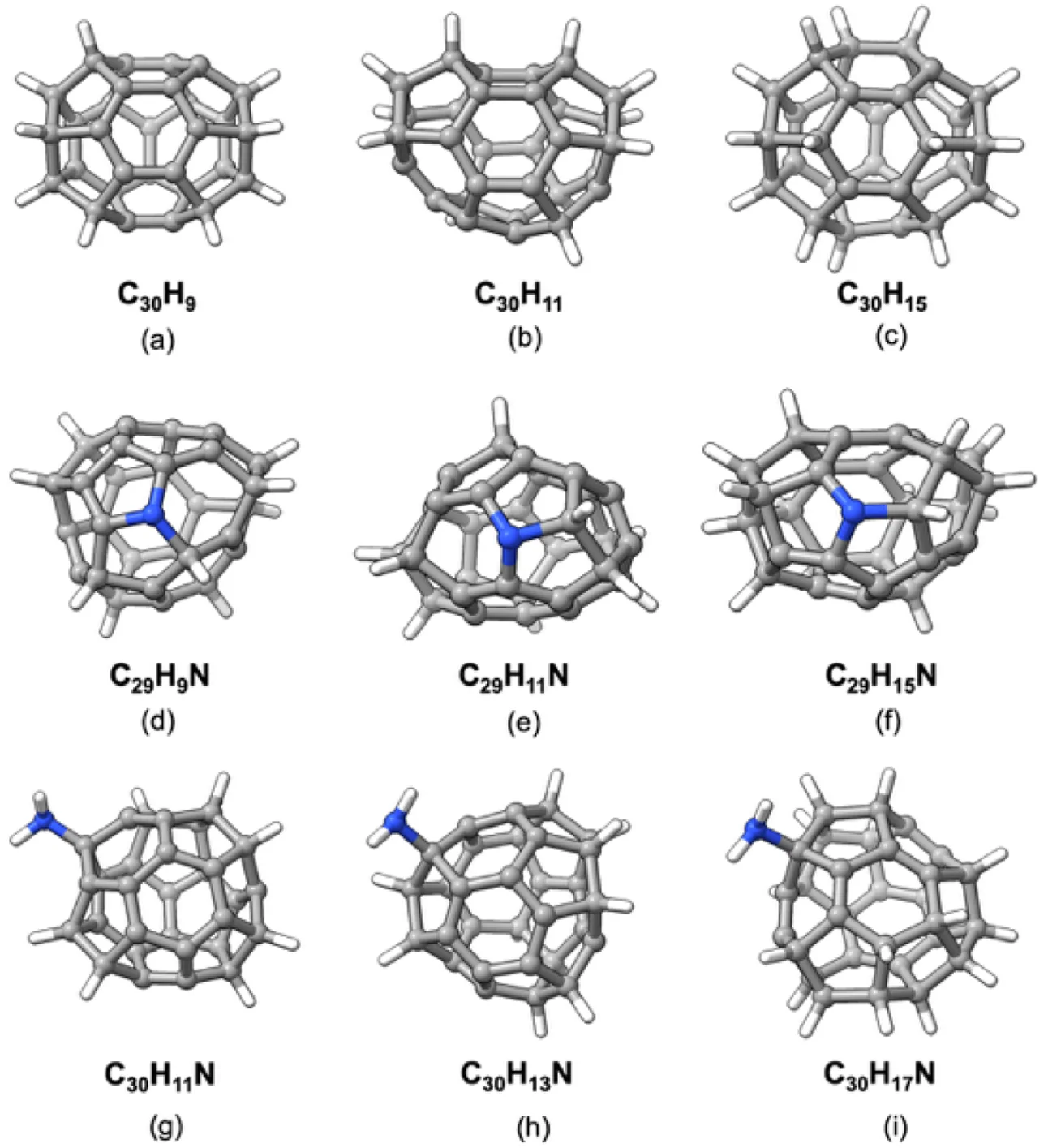
Representative lowest-energy structures of the partially hydrogenated C_30_ carbon clusters considered in this study, including pristine (a, b and c), nitrogen-doped (d, e and f), and NH_2_-functionalized (g, h and i) systems at increasing hydrogenation levels. We remark that the absorption spectra reported below correspond to averages over the five selected isomers for each composition, and that the geometries shown here are those of the most stable isomer.

**Figure 6:**
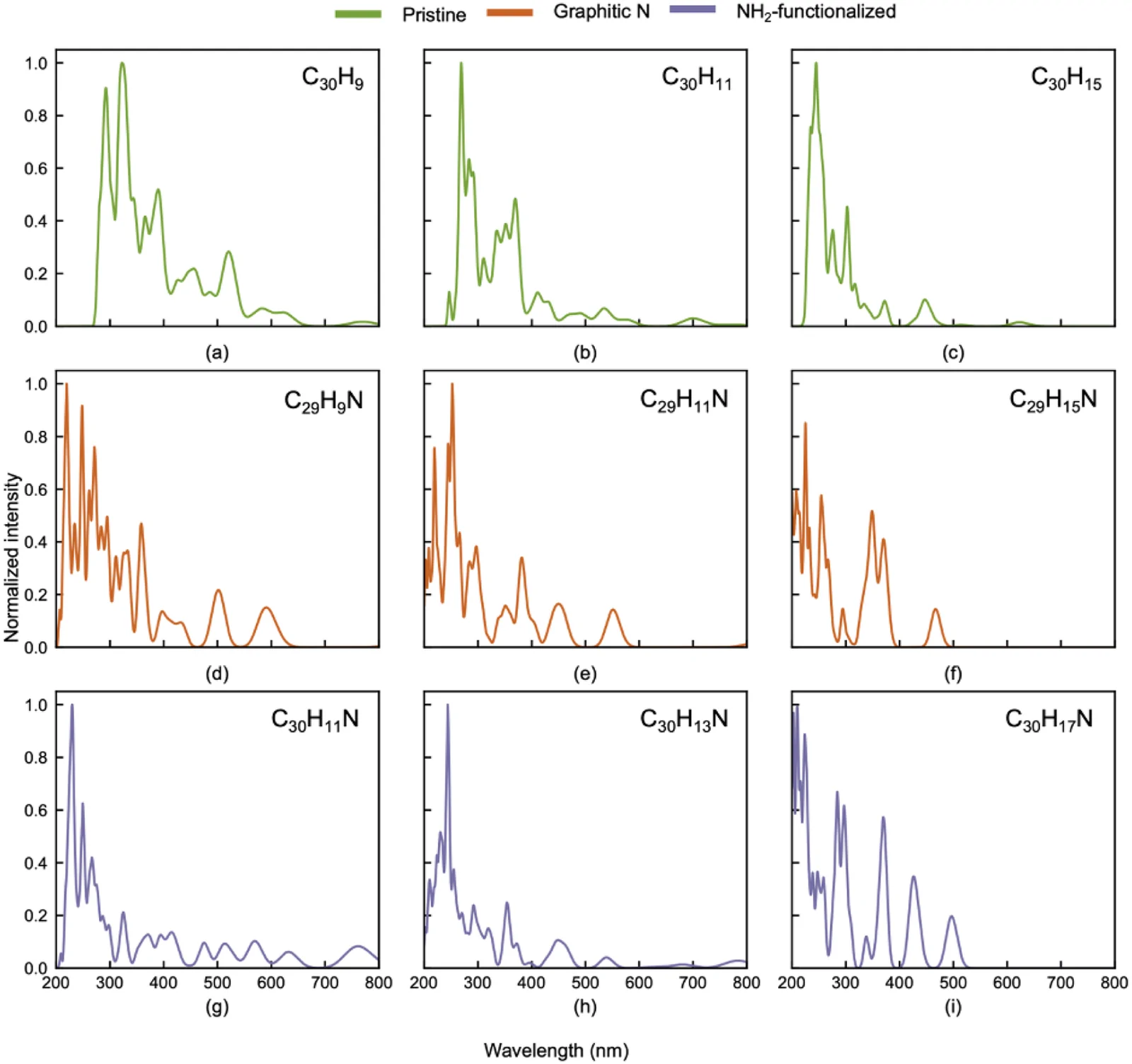
Calculated absorption spectra of partially hydrogenated C_30_ based carbon clusters as a function of hydrogenation degree and doping/functionalization. Moving from left to right corresponds to increasing hydrogenation of the parent carbon framework, while the three rows correspond to the pristine, nitrogen-doped, and NH_2_-functionalized chemical classes. Panels (a–c) show the pristine clusters C_30_H_9_, C_30_H_11_, and C_30_H_15_; panels (d–f) show the corresponding nitrogen-doped systems C_29_H_9_N, C_29_H_11_N, and C_29_H_15_N; and panels (g–i) show the corresponding NH_2_-functionalized systems C_30_H_11_N, C_30_H_13_N, and C_30_H_17_N. Each spectrum represents the average absorption profile obtained from the five selected isomers of the corresponding system. Intensities are normalized to allow a direct comparison of the spectral profiles.

For all three classes of systems, increasing the hydrogen content (from left to right in Figure 6), going from 9H to 15H structures, results in a progressive redistribution of the spectral weight toward lower wavelengths, accompanied by a progressive disappearance of low-energy absorption features. This behavior is indicative of a widening of the optical gap, which can be expected from basic chemistry and attributed to the progressive disruption of the delocalized π-network upon hydrogenation and the concomitant increase in sp^3^ character.

At low hydrogenation levels (9H based structures), all systems display relatively broad and structured absorption profiles extending well into the visible region, consistent with the presence of delocalized π → π* excitations. As the hydrogen content increases, the spectra become sharper and with more intense features in the UV region (≈200–350 nm), while the visible-range absorption is suppressed. This trend suggests a transition from collective, delocalized excitations to more localized electronic transitions associated with smaller conjugated domains.

The effect of graphitic nitrogen incorporation can be had by comparing the spectra in the first row with those in the second. Nitrogen-doped systems (second row) exhibit a broader distribution of absorption bands in the UV region and a partial enhancement of low-energy transitions that survives for high hydrogenation levels. This indicates that nitrogen introduces additional low-lying electronic states, likely with partial n-character, which contribute to maintaining absorption channels at lower energies despite the reduced conjugation.

An even more pronounced modification is observed for NH_2_-functionalized clusters (bottom row). In these systems, the spectra display more structured and, in some cases, red-shifted features compared to the corresponding pristine analogues, particularly at intermediate and high hydrogenation levels. This behavior can be attributed to the active role of the amine group, whose lone pair can participate in low-energy excitations, leading to mixed π–n transitions and enhanced localization of the excited states.

Overall, the combined effect of hydrogenation and nitrogen-based functionalization results in a delicate balance between conjugation extension, electronic localization, and the emergence of heteroatom-induced states. These factors collectively determine both the position and the shape of the absorption spectra, providing an intricate framework to rationalize the observed trends across the different chemical classes.

Overall, increasing hydrogen content produces a progressive blue-shift of the absorption bands, but the presence of graphitic nitrogen gives rise to an increase of the intensity of the spectrum at larger wavelengths. While hydrogenation alone leads to a systematic depletion of low-energy absorption, nitrogen incorporation (particularly in the form of graphitic functionalization) partially counteracts this effect by introducing and stabilizing localized electronic states.

Figure 7 compares the spectra obtained at a fixed hydrogenation level of H11 (the corresponding spectra for H9 and H15 are shown in Figure S4); each spectrum represents an average over the five selected isomers for each composition. The overall geometry of the three systems, whose global minima are reported in Figure 5, is very similar, allowing us to isolate the role of nitrogen doping in modifying the electronic properties from geometric effects. An apparent blue-shift is observed for nitrogen-containing systems, which contrasts with previous findings on unsaturated clusters, where graphitic nitrogen consistently induced a red-shift of the absorption bands.^20^ This discrepancy may stem from the competing effects of nitrogen and hydrogen, whose balance is not straightforward to assess.

**Figure 7:**
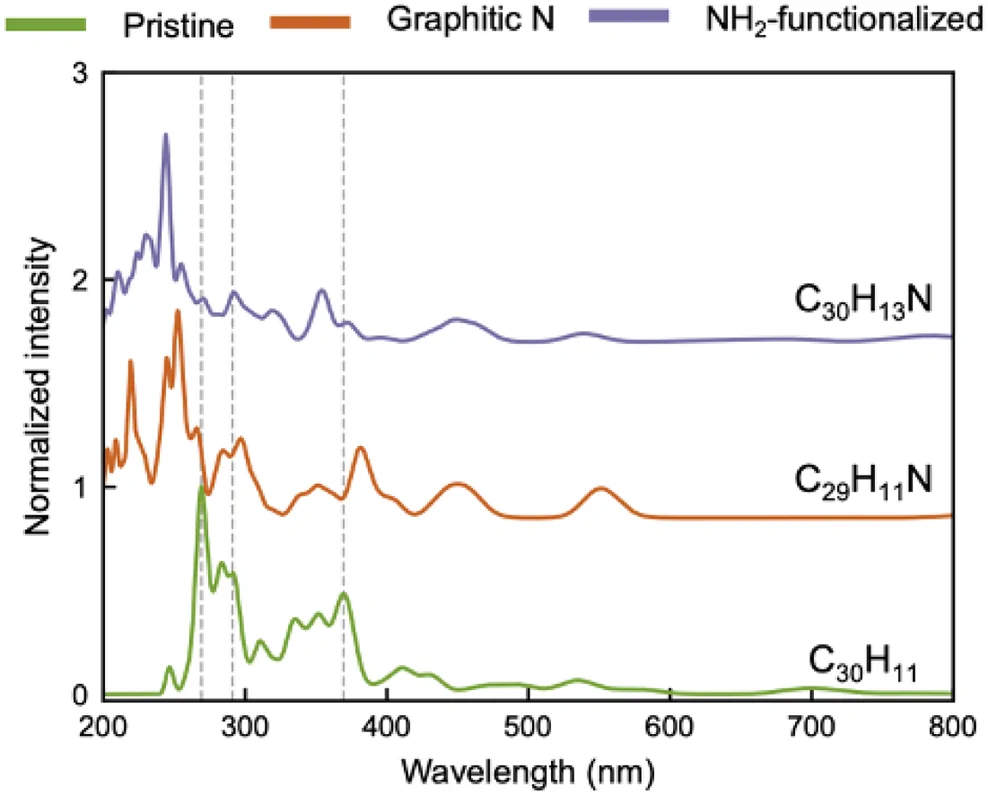
Effect of chemical composition on the absorption spectra of partially hydrogenated carbon clusters at fixed hydrogenation level (H11). The spectra correspond to pristine (C_30_H_11_), nitrogen-doped (C_29_H_11_N), and NH_2_-functionalized (C_30_H_13_N) clusters and represent averages over the five selected isomers for each composition. Each spectrum is normalized to its maximum intensity and vertically shifted for clarity. The dashed vertical lines indicate the positions of the three most intense absorption bands of the pristine cluster, identified from the maxima of the convoluted spectrum, and serve as visual guides to track changes induced by chemical modification.

In order to disentangle the complexity arising from the two competing and counteracting effects of N-doping and hydrogenation, we have computed three quantitative descriptors derived from the ensemble-averaged C_30_-based absorption spectra of Figure 6. The first descriptor is the absorption spectral centroid, ⟨*E*⟩, evaluated over the 1.5–6.0 eV range. The second is the low-energy visible spectral weight, *W*_vis_, defined as the fraction of the integrated absorption intensity between 1.5 and 3.1 eV relative to that between 1.5 and 6.0 eV. Finally, we quantify the similarity between pristine and graphitic-N spectra at the same H/C ratio through the normalized spectral overlap *S*, a normalized cosine similarity between the two spectra, where *S* = 1 corresponds to identical spectral shapes.

The resulting trends are shown in Figure 8 and numerically in Table 2. The magnitude of the difference in absorption centroid between pristine and graphitic-N systems decreases monotonically from 1.16 eV in the unsaturated limit to 0.99, 0.69, and finally 0.08 eV at H/C = 0.30, 0.37, and 0.50, respectively. A similarly pronounced convergence is observed for the visible spectral weight: |Δ*W*_vis_| decreases from 0.346 at H/C = 0 to 0.102, 0.018, and 0.004 with increasing hydrogenation. Consistently, the normalized spectral overlap increases from 0.18 in the unsaturated systems to 0.43, 0.44, and 0.55 over the same sequence. Although in unsaturated clusters graphitic nitrogen has a strong red-shifting effect, the progressive saturation counterbalances this effect to the point that the N-doped saturated clusters have absorption markers that are almost indistinguishable from the saturated pristine counterparts. Even a modest degree of hydrogenation seems sufficient to counteract the red-shifting effect of graphitic nitrogen. In both cases, saturation essentially suppresses absorption in the visible range. An additional feature revealed by this analysis is that the sign of the centroid shift changes between the unsaturated and the first hydrogenated regime, from negative at H/C = 0 to positive for H/C ≥ 0.30. Thus, the initial hydrogenation step deeply reorganizes the underlying *π*-electronic response.

**Figure 8:**
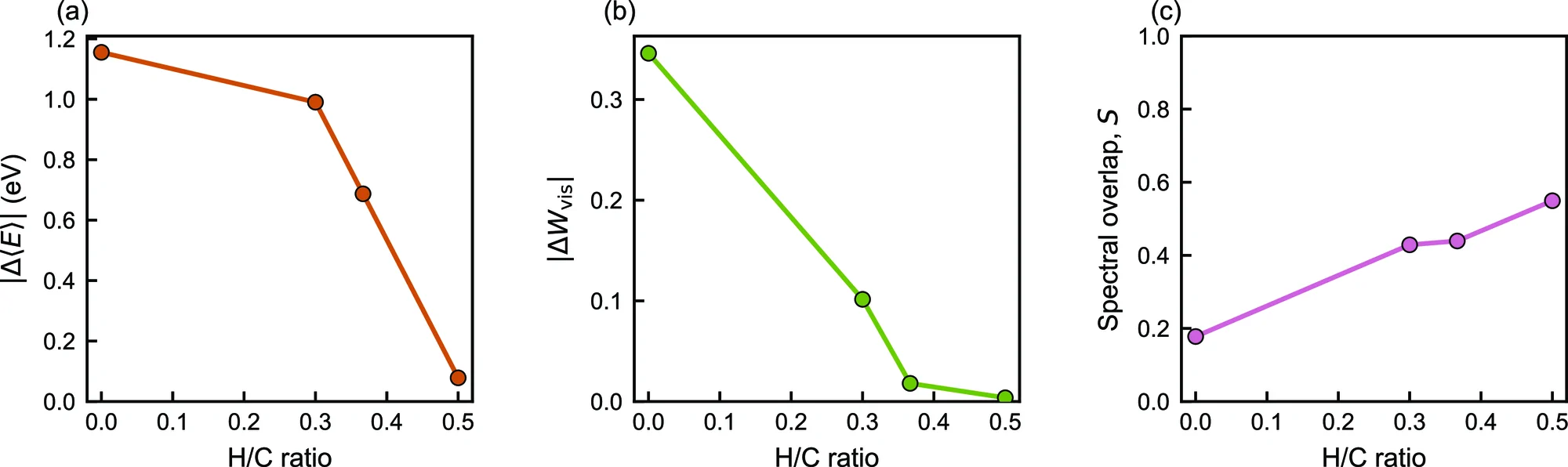
Quantitative convergence of the absorption response of pristine and N-doped C_30_-based systems with increasing hydrogenation. (a) Absolute difference in the absorption spectral centroid, |Δ⟨*E*⟩|; (b) absolute difference in the visible spectral weight, |ΔW_vis_|; and (c) normalized spectral overlap, S, between the ensemble-averaged pristine and graphitic-N absorption spectra.

**Table 2:** Quantitative Optical Descriptors Derived from the Ensemble-Averaged Absorption Spectra of Pristine and Graphitic-N C_30_-Based Systems as a Function of H/C Ratio^a^

H/C	⟨*E*⟩ (eV) pristine	⟨*E*⟩ (eV) N-doped	Δ⟨*E*⟩ (eV)	W_vis_ pristine	W_vis_ N-doped	|ΔW_vis_|	S (pristine, graphitic N)
0.0000	4.2229	3.0678	–1.1552	0.0867	0.4327	0.3459	0.1779
0.3000	3.6449	4.6352	+0.9903	0.1707	0.0692	0.1015	0.4289
0.3667	4.0128	4.6999	+0.6871	0.0848	0.0667	0.0180	0.4393
0.5000	4.7532	4.8316	+0.0784	0.0260	0.0223	0.0037	0.5494

a See text for definitions.

In other words, hydrogenation and graphitic-N substitution act as competing perturbations of the same conjugated carbon framework. In the unsaturated structures, the extended *π* network allows the heteroatom to induce a strong redistribution of low-energy oscillator strength. Progressive hydrogenation disrupts and localizes this network, reducing the spatial extent over which the N-induced perturbation can act. As a consequence, the pristine and graphitic-N optical responses progressively converge at high hydrogenation.

In Figure 9, we report the absorption spectra of pristine (green) and nitrogen-doped (orange) clusters at different hydrogenation levels together with their non hydrogenated counterpart (already presented in our previous work^20^) thus highlighting the effect of heteroatom incorporation.

**Figure 9:**
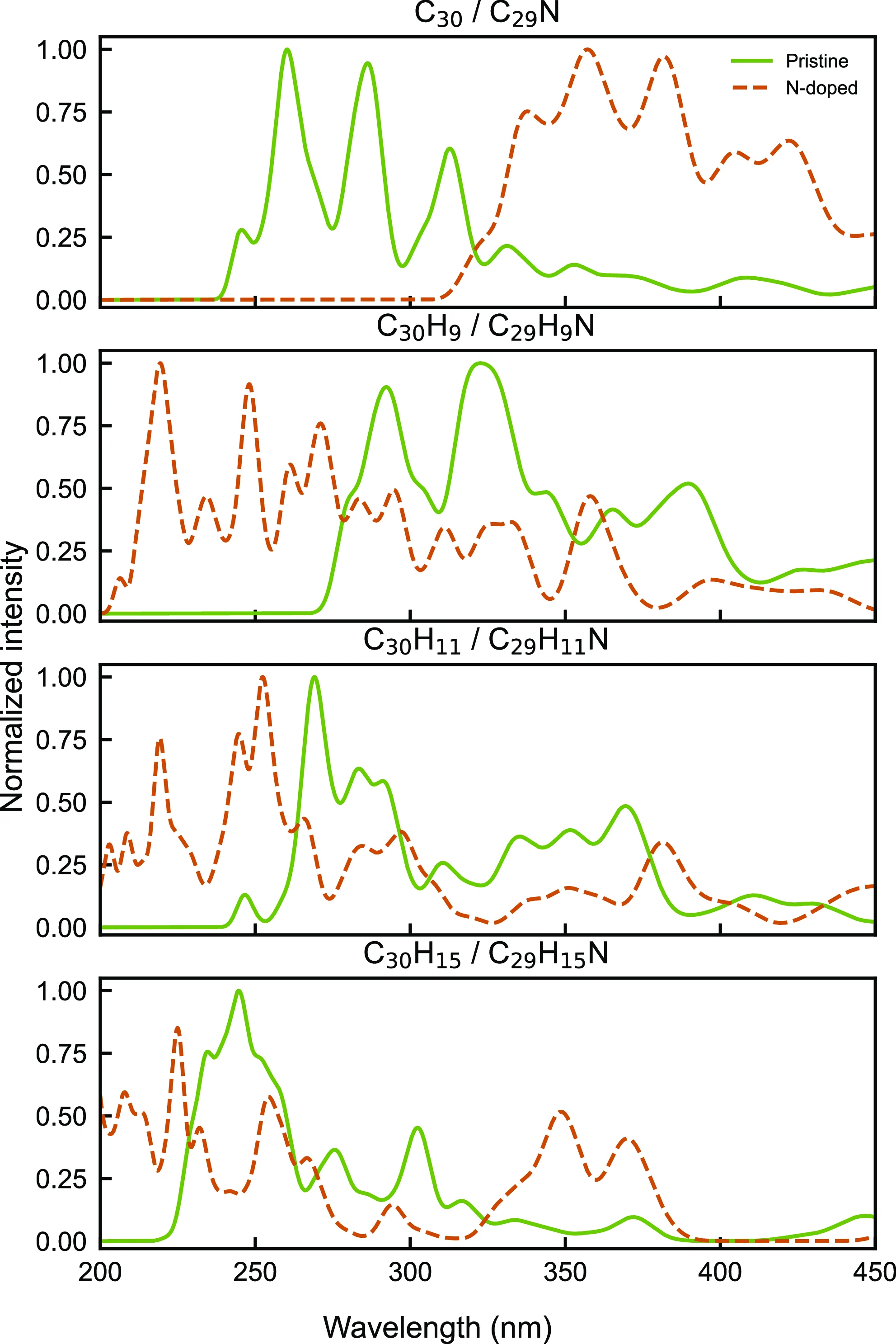
Comparison of the calculated absorption spectra of pristine (green) and nitrogen-doped (orange) carbon clusters at increasing hydrogenation levels. For each system, spectra are obtained by averaging the TD-DFT absorption profiles of five selected isomers and subsequently normalized to their maximum intensity. The top panel shows the unsaturated clusters (C_30_ and C_29_N), followed from top to bottom by progressively hydrogenated systems.

In the absence of hydrogenation (top panel), the difference between C_30_ and C_29_N is extremely pronounced: the N-doped system exhibits a substantial redistribution of spectral weight toward lower energies and a markedly different spectral profile, reflecting the strong perturbation that nitrogen incorporation introduces into the delocalized π-electron network of a fully conjugated system. Upon partial hydrogenation, the spectral profiles begin to show some degree of overlap, although significant differences persist: the pristine cluster retains more structured features at higher energies, while the N-doped system maintains broader and more intense absorption in the low-energy region, suggesting that nitrogen-induced states remain active despite the initial disruption of conjugation. As hydrogenation progresses to H11, the distinction between pristine and doped systems becomes less pronounced, with the main absorption features aligning more closely in energy, though differences in intensity distribution persist. This trend reflects the progressive fragmentation of the π-network, which reduces the relative impact of nitrogen on the global electronic structure. At high hydrogenation (H15), the spectra of pristine and nitrogen-doped clusters become similar in both peak positions and overall shape. In this regime, the electronic structure is dominated by localized states associated with small, conjugated domains, and the perturbation introduced by a single nitrogen atom becomes insufficient to significantly alter the overall absorption profile.

Taken together, these results demonstrate that the influence of nitrogen doping is strongly modulated by the degree of conjugation of the carbon framework: while nitrogen acts as a significant perturbation in extended π-systems, its relative impact progressively diminishes as hydrogenation localizes the electronic structure.

Having discussed the absorption properties, we now turn to emission, whose computational characterization is intrinsically more challenging. Unlike vertical absorption, the calculation of emission requires the description of excited-state relaxation and therefore relies on additional assumptions concerning the relevant emissive states and their optimized geometries. Consequently, the reliability of the predicted emission properties depends not only on these assumptions but turns out to be more sensitive to the approximations intrinsic to the TD-DFT methodology employed.

We present plausible fluorescence spectra for three systems at the fixed H/C ratio of 0.30 in Figure 10 where we report the same spectra both in terms of wavelength and energy. For the pristine C_30_H_9_ cluster (panels a), the Stokes shift is relatively large (ΔE = 3.14 eV), reflecting a pronounced structural and electronic reorganization upon relaxation of the excited states. These data are compatible with the most prominent feature of the emissive CNDs with absorption in the UV range and typical emissions in the green visible region. Here, due to smaller system sizes, the emission appears in the red limit of the visible spectrum.

**Figure 10:**
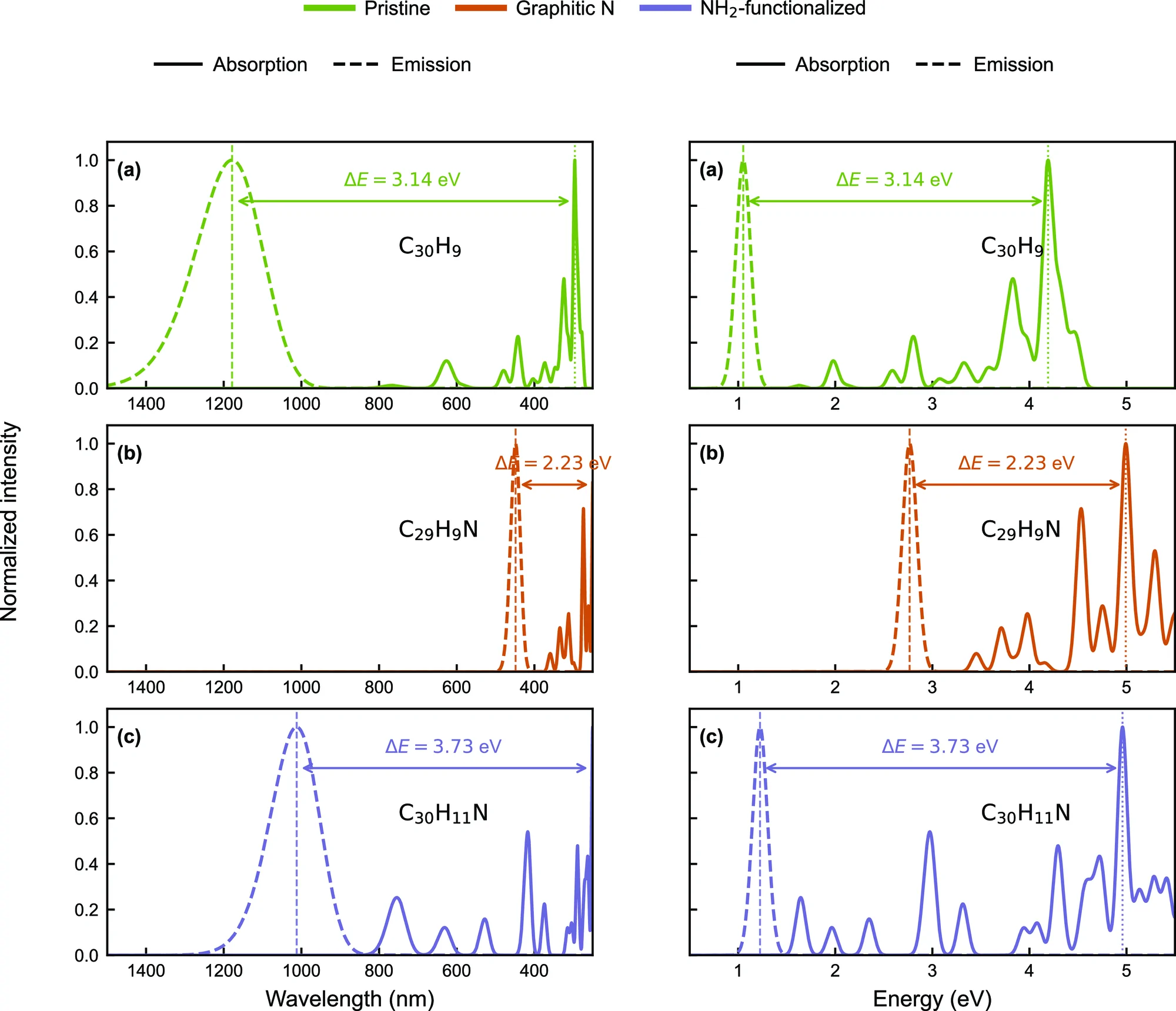
Simulated absorption (solid lines) and emission (dashed lines) spectra of C_30_ based systems with an H/C ratio of 0.30, plotted as a function of both wavelength (nm, left panels) and photon energy (eV, right panels). Panels (a) show the pristine C_30_H_9_ system, panels (b) the nitrogen-doped C_29_H_9_N system, and panels (c) the NH_2_-functionalized C_30_H_11_N system. Absorption spectra were computed at the ground-state geometry, while emission spectra were obtained from vertical transitions at the optimized excited-state minimum of each system i.e., D_1_ for (a), S_1_ for (b), and S_2_ for (c). Gaussian broadening was applied to all transitions, with fwhm values of 0.12 eV for absorption and 0.18 eV for emission. Arrows indicate the corresponding Stokes shifts.

A markedly different behavior is observed for the nitrogen-doped system (C_29_H_9_N, panels b), where the Stokes shift is significantly reduced (ΔE = 2.23 eV). This suggests that the excited-state relaxation is less pronounced, which can be attributed to the presence of nitrogen-induced electronic states that partially localize the excitation already at the Franck–Condon region. As a result, the reorganization required to reach the emissive state is reduced, leading to a smaller energy difference between absorption and emission.

In contrast, the NH_2_-functionalized system (C_30_H_11_N, panels c) exhibits the largest Stokes shift (ΔE = 3.73 eV), indicating an even stronger degree of excited-state relaxation. This behavior can be rationalized by considering the active role of the amine group, whose lone pair contributes to low-energy excitations with significant charge redistribution, as explained below. Upon excitation, this leads to highly localized excited states that undergo substantial relaxation, resulting in a pronounced stabilization of the emissive state.

Overall, these results highlight that the magnitude of the Stokes shift is strongly dependent on the nature of the electronic excitation and its degree of localization. While pristine systems exhibit large shifts due to global π-electron reorganization, nitrogen doping reduces this effect by prelocalizing the excitation, whereas NH_2_ functionalization enhances excited-state relaxation through the formation of strongly localized π–n states. This demonstrates that heteroatom incorporation provides an effective strategy to tune not only absorption energies but also emission properties and relaxation pathways in carbon-based clusters.

Considering the positions of the emission bands, in our models, the nitrogen-doped system C_29_H_9_N exhibits a well-defined emission band in the visible region, indicating a radiatively active excited state. In contrast, the pristine and NH_2_-functionalized clusters show emission intensity at short wavelengths, consistent with very small energy gaps and weak oscillator strengths, suggesting that these clusters can present important nonradiative relaxation pathways.

It would be useful to compare our results directly with those reported for small carbon clusters. Unfortunately, the literature on these specific doping and functionalization strategies remains scarce, leaving us without adequate reference data for a direct comparison. We therefore considered suitable experimentally accessible carbon-based materials as proxies.

Table 3 compares selected calculated optical properties of representative cluster systems with those of the corresponding experimental proxies. The comparison is necessarily qualitative because of the differences between the systems involved, but nevertheless provides a useful phenomenological benchmark for assessing the physical relevance of the models. Amorphous carbon films and GQDs were excluded from Table 3 because their optical properties are not directly comparable with those of the small molecular clusters studied here. For amorphous carbon films, the relevant quantity is generally the optical gap, whereas GQD spectra are strongly affected by their larger size and structural complexity.

**Table 3:** Comparison between the Calculated Optical Properties of Representative C_30_-Based Clusters and Experimental Values Reported for Related Carbon-Based Systems

**Present work**				**Experimental proxy**			
System	Abs. max (eV)	Em. max (eV)	Stokes shift (eV)	Proxy	Abs. max. (eV)	Em. max. (eV)	Stokes shift (eV)
C_30_H_9_	4.19	1.05	3.14	Coronene C_24_H_12_ ^47^	∼4.07	∼2.78	∼1.29
C_29_H_9_N	4.99	2.76	2.23	N-rich carbon nanodots^44^	3.10	2.40	0.70
C_30_H_11_N	4.96	1.23	3.73	Amine-rich carbon nanodots^46^	4.34	3.48	0.85

The absorption energy of the pristine cluster is compared with that of coronene, while the optical properties of the cluster incorporating graphitic nitrogen are compared with those of N-rich CNDs, and those of the NH_2_-functionalized cluster with amine-rich CNDs. For the pristine cluster, and those functionalized with amino group, the absorption energy is in good agreement with their respective experimental proxy, whereas they exhibit a much larger Stokes shift, likely due to the presence of low-lying electronic states involved in the emission process.

For the N-doped cluster, the calculated absorption is significantly blue-shifted relative to that of N-rich CNDs, an effect that can reasonably be attributed to the much smaller size of the model. Studies on nitrogen-doped carbon nanodots (N-CDs), which represent more extended and functionalized carbon frameworks, consistently report emission in the visible range, typically between ∼2.1 and 3.0 eV (≈400–600 nm). In these systems, the emission is generally broad and excitation-dependent and has been associated with a manifold of surface-localized states related to nitrogen-containing functional groups.^44^ Despite these similarities in the emission energy range, it is important to note that carbon nanodots typically exhibit fluorescence dominated by surface or defect states, often resulting in broad, excitation-dependent spectra.^45^ In contrast, the emission observed in the present C_29_H_9_N cluster is associated with a discrete electronic transition, reflecting its molecular character. Therefore, the nitrogen-doped cluster can be viewed as an intermediate system, where the introduction of a heteroatom is sufficient to activate radiative decay channels within an otherwise nonemissive carbon framework, while still preserving a well-defined, molecule-like emission.

Similarly, amine-rich N-doped carbon nanodots have been shown to exhibit tunable emission spanning from the near-UV to the visible region (≈2.6–3.5 eV), strongly influenced by surface passivation and the presence of NH_2_ functionalities.^46^ These observations highlight the key role of nitrogen in introducing new emissive states and modulating the nature and energy of the emissive states.

To clarify the nature of the electronic excitations, natural transition orbitals (NTOs) were examined for representative absorption and emission transitions of the C_30_ family. The orbitals are reported in Figure 11 with additional details and specific numbers in Table S5.

**Figure 11:**
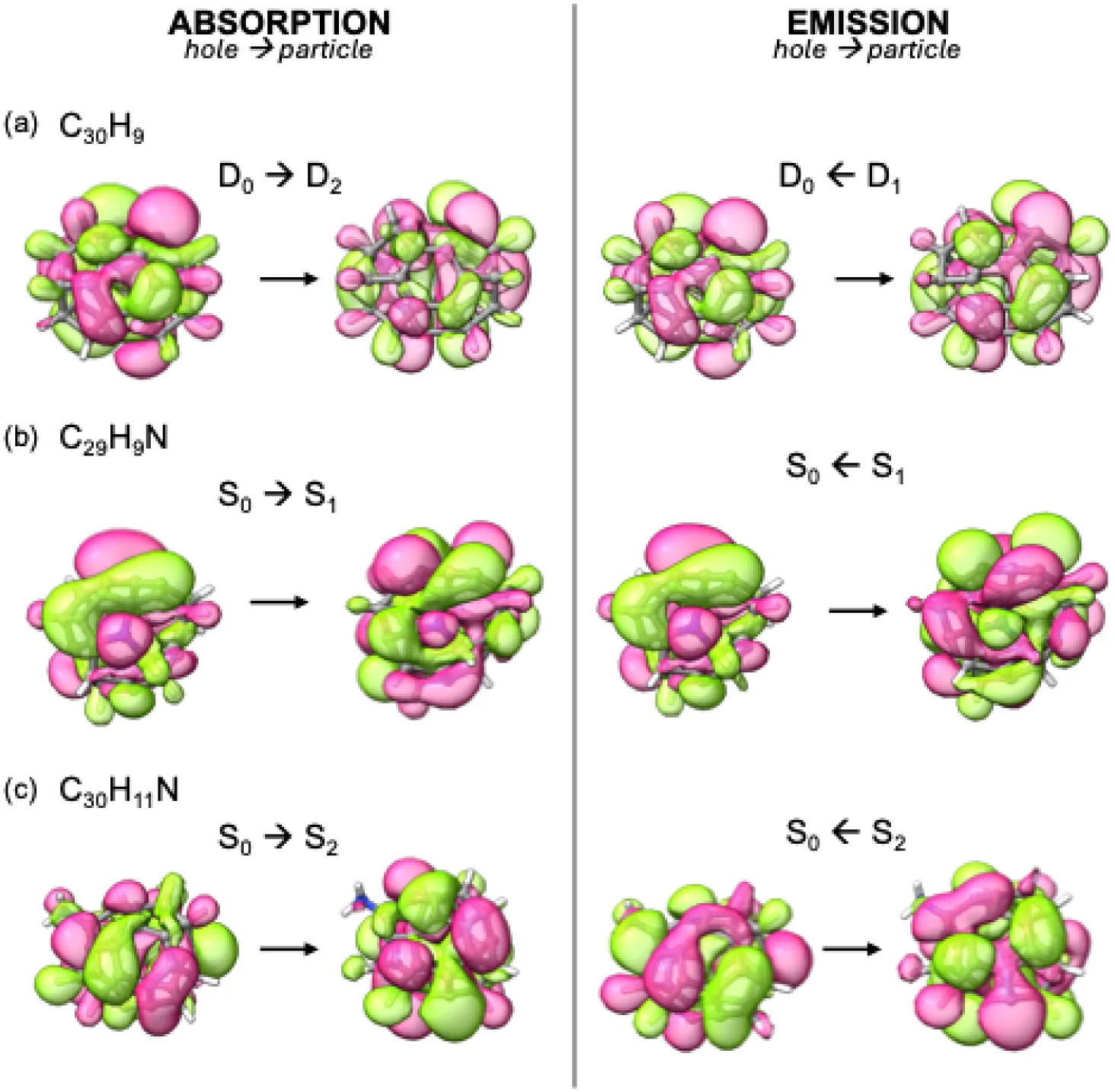
Dominant hole–particle natural transition orbital (NTO) pairs associated with representative absorption and emission transitions of the three C_30_ based systems at H/C = 0.30: (a) pristine C_30_H_9_, (b) graphitic-N C_29_H_9_N, and (c) NH_2_-functionalized C_30_H_11_N. The specific transitions are indicated on top of the orbital pairs. Those for emission are computed at the excited-state minimum, D_1_ for C_30_H_9_ and S_1_ for C_29_H_9_N and S_2_ for C_30_H_11_N. The same isovalue (±0.01 au) and molecular orientation were used to facilitate direct comparison.

For the pristine system C_30_H_9_, the dominant NTOs are distributed over a large portion of the carbon framework and exhibit substantial spatial overlap, supporting the assignment of the excitation as predominantly local, delocalized π → π* in character. Nitrogen substitution and amino functionalization modify the spatial distribution of the hole and particle orbitals, but no pronounced long-range separation is evident from the NTOs, suggesting that the corresponding excitations retain a substantial local character rather than exhibiting strong charge-transfer behavior.

Quantitative transition-density analysis (Table S5) further supports this interpretation for the two restricted N-containing systems. For C_29_H_9_N, the selected absorption and emission states exhibit only modest charge-transfer contributions of approximately 7–11% between fragments,^43^ where fragment 1 corresponds to the only graphitic nitrogen atom, while fragment 2 is the rest of the carbon-based framework. The dominant directional component corresponds to charge redistribution from the N-centered fragment toward the remaining cluster framework. By contrast, the NH_2_-functionalized cluster displays only approximately 1–2% interfragment charge-transfer character for both absorption and emission, demonstrating that its low-energy excitations remain framework-centered. Here, fragments 1 is identified with the −NH_2_ group, while fragment 2 is the rest of the cluster.

The dominant NTO shapes are also largely preserved upon excited-state relaxation. Consistently, excited-state optimization produces only minimal structural reorganization, with changes in geometries upon excited state relaxation that are relatively small with RMSDs of ∼0.04 Å for all clusters. These values confirm the rigidity of the carbon frameworks and indicate that the absorption–emission energy separation is not associated with large-scale geometric distortion, but instead primarily reflects electronic-state relaxation and the involvement of different excited states in absorption and emission.

## 4 Summary and Conclusions

In this work, we have investigated the structural and optical evolution of medium-sized carbon clusters (C_20_–C_35_) as a function of hydrogenation and nitrogen-based doping and functionalization. From a structural perspective, hydrogenation is an important factor controlling the configurational landscape, inducing a progressive transition from conjugated frameworks toward more localized and distorted structures. This effect is captured by the analysis of the structure in terms of geometric features that shows how hydrogen content drives the primary structural variability, while nitrogen incorporation introduces a less discriminating effect without fundamentally altering the global shape.

Energetically, hydrogenation leads to an evident decrease in atomization energy per atom, reflecting the disruption of π-delocalization and increasing bond distortion. This destabilizing effect is more pronounced in larger clusters, where extended conjugation plays a more important stabilizing role. Nitrogen doping and amino functionalization further reduce stability, because of the effect of heteroatoms on the carbon framework.

The optical response of the clusters is strongly correlated with these structural changes. Increasing hydrogenation results in a clear blue shift and suppression of low-energy absorption, consistent with the fragmentation of conjugated domains. In contrast, nitrogen incorporation—particularly in the form of a −NH_2_ group—introduces additional electronic states that partially restore low-energy absorption, thus indicating a contrasting role between hydrogenation and insertion of heteroatoms in modulating the electronic structure.

Excited-state analysis reveals that emission properties are highly sensitive to the nature of the electronic excitation. Pristine clusters exhibit large Stokes shifts associated associated with electronic-state relaxation and the involvement of different excited states while structural reorganization plays a limited role. Nitrogen-doped systems, show more localized excitations and reduced reorganization energies, leading to more efficient and well-defined emission in the visible region. NH_2_-functionalized clusters display large Stokes shifts, reflecting pronounced excited-state localization. Taken together, these results highlight that the optical behavior of carbon clusters is governed by a delicate balance between hydrogenation-induced localization and heteroatom-induced electronic states. This work suggests a simple and unified picture that connects structural disorder, chemical composition, and photophysical response, offering possibly important insights for the design of carbon-based nanomaterials, including carbon nanodots and other hydrogenated amorphous carbon systems.

## Supplementary Material



## Notes

Table S1 and Table S2: excited states selection for relaxation and emission calculations; Table S3: candidate emissive states for pristine and functionalized species; Figure S1: atomization energy; Figure S2: structural descriptors for PCA analysis and their description; Figure S3: calculated absorption spectra for all clusters; Figure S4: absorption spectra comparison across compositions; Table S5: quantitative indicators of selected electronic transitions (PDF)

## References

[1] Contreras C. S., Salama F. (2013). Laboratory Investigations of Polycyclic Aromatic Hydrocarbon
Formation and Destruction in the Circumstellar Outflows of Carbon
Stars. Astrophys. J., Suppl. Ser..

[2] Lagana S., La Rocca A., Cairns A., Fay M. W., Apicella B., Ciajolo A., Russo C. (2023). 2D and 3D
TEM Characterisation of
Benzene and Ethylene Soot. Fuels.

[3] Zhang Y., Kim D., Rao L., Zhang R., Kook S., Kim K. S., Kweon C.-B. (2017). The Soot
Particle Formation Process inside the Piston
Bowl of a Small-Bore Diesel Engine. Combust.
Flame.

[4] Rao L., Zhang Y., Kook S., Kim K. S., Kweon C.-B. (2020). Morphology
and Internal Structure of Soot Particles under the Influence of Jet–Swirl
and Jet–Jet Interactions in a Diesel Combustion Environment. Combust. Flame.

[5] Russo C., Apicella B., Ciajolo A. (2023). Hydrogen Sp^2^ Carbon Hybridization,
and Sp^2^ Clustering as Pieces of the Puzzling Nanostructure
of Soot: A Closer Look. Energy Fuels.

[6] Pino T., Chabot M., Béroff K., Godard M., Fernandez-Villoria F., Le K. C., Breuer L., Herder M., Wucher A., Bender M., Severin D., Trautmann C., Dartois E. (2019). Release of Large Polycyclic Aromatic Hydrocarbons and
Fullerenes by Cosmic Rays from Interstellar Dust: Swift Heavy Ion
Irradiations of Interstellar Carbonaceous Dust Analogue. Astron. Astrophys..

[7] Sandford S. A., Nuevo M., Bera P. P., Lee T. J. (2020). Prebiotic Astrochemistry
and the Formation of Molecules of Astrobiological Interest in Interstellar
Clouds and Protostellar Disks. Chem. Rev..

[8] Tielens A. G. G. M. (2008). Interstellar
Polycyclic Aromatic Hydrocarbon Molecules. Annu.
Rev. Astron. Astrophys..

[9] Berné O., Tielens A. G. G. M. (2012). Formation
of Buckminsterfullerene (C_60_)
in Interstellar Space. Proc. Natl. Acad. Sci.
U. S. A..

[10] Siebenmorgen R., Heymann F., Chini R. (2025). Luminosity Distance and Extinction
by Submicrometer-Sized Grains. Astrophys. J.
Lett..

[11] Herrero V. J., Jiménez-Redondo M., Peláez R. J., Maté B., Tanarro I. (2022). Structure and Evolution of Interstellar
Carbonaceous Dust. Insights from the Laboratory. Front. Astron. Space Sci..

[12] Zhou Z., Sfeir M. Y., Zhang L., Hybertsen M. S., Steigerwald M., Brus L. (2006). Graphite Tubular PAHs, and the Diffuse
Interstellar Bands. Astrophys. J..

[13] Pla P., Dubosq C., Rapacioli M., Posenitskiy E., Alcamí M., Simon A. (2021). Hydrogenation of C_24_ Carbon
Clusters: Structural Diversity and Energetic Properties. J. Phys. Chem. A.

[14] Sciortino L., Sciortino A., Popescu R., Schneider R., Gerthsen D., Agnello S., Cannas M., Messina F. (2018). Tailoring
the Emission Color of Carbon Dots through Nitrogen-Induced Changes
of Their Crystalline Structure. J. Phys. Chem.
C.

[15] Essner J. B., Kist J. A., Polo-Parada L., Baker G. A. (2018). Artifacts and Errors
Associated with the Ubiquitous Presence of Fluorescent Impurities
in Carbon Nanodots. Chem. Mater..

[16] Ehrat F., Bhattacharyya S., Schneider J., Löf A., Wyrwich R., Rogach A. L., Stolarczyk J. K., Urban A. S., Feldmann J. (2017). Tracking the Source of Carbon Dot
Photoluminescence: Aromatic Domains versus Molecular Fluorophores. Nano Lett..

[17] Alafeef M., Srivastava I., Aditya T., Pan D. (2024). Carbon Dots: From Synthesis
to Unraveling the Fluorescence Mechanism. Small.

[18] Đorđević L., Arcudi F., Cacioppo M., Prato M. (2022). A Multifunctional Chemical
Toolbox to Engineer Carbon Dots for Biomedical and Energy Applications. Nat. Nanotechnol..

[19] D’ambrosio F., Frustaci A., Bodo E. (2025). A Bottom-up Computational
Study of
Doped Amorphous Carbon Clusters as Precursors of Carbon Nanodots. J. Phys. Chem. A.

[20] D’ambrosio F., Frustaci A., Azzali A., Bodo E. (2026). Investigating the Absorption
Properties of Pure and Nitrogen-Doped Carbon Clusters as Models for
the Core of Carbon Nanodots. J. Phys. Chem.
A.

[21] Bennedjai S. C., Hammoutène D., Senent M. L. (2019). Theoretical Characterization
of C_3_ H and C_5_ H and Their Anions. Astrophys. J..

[22] Robertson J., O’reilly E. P. (1987). Electronic and Atomic Structure of
Amorphous Carbon. Phys. Rev. B.

[23] Rusli
Robertson R., Robertson G. A. J., Amaratunga G. A. J. (1997). Photoluminescence
in Hydrogenated Amorphous Carbon. Diamond Relat.
Mater..

[24] Strelnikov D. V., Link M., Weippert J., Kappes M. M. (2019). Optical Spectroscopy
of Small Carbon Clusters from Electron-Impact Fragmentation and Ionization
of Fullerene-C _60_. J. Phys. Chem.
A.

[25] Hong X., Su L., Li J. (2024). Theoretical Investigation
on the Optical Absorption
Spectra in Cyclo­[n]­Carbons (n = 10, 14, 18). Chem. Phys. Lett..

[26] Dubosq C., Pla P., Dartois E., Simon A. (2023). Spectroscopic
Investigation of Interstellar
Hydrogenated Carbon Clusters: Infrared and Optical Spectra of C_24_ H_n_ Populations. Astron.
Astrophys..

[27] Dubosq C., Calvo F., Rapacioli M., Dartois E., Pino T., Falvo C., Simon A. (2020). Quantum Modeling
of the Optical Spectra
of Carbon Cluster Structural Families and Relation to the Interstellar
Extinction UV Bump. Astron. Astrophys..

[28] Bond T. C., Bergstrom R. W. (2006). Light Absorption
by Carbonaceous Particles: An Investigative
Review. Aerosol Sci. Technol..

[29] De
Souza B. (2025). GOAT: A Global Optimization Algorithm for Molecules and Atomic Clusters. Angew. Chem., Int. Ed..

[30] Neese F. (2025). Software Update:
The ORCA Program System—Version 6.0. WIREs Comput. Mol. Sci..

[31] Bannwarth C., Ehlert S., Grimme S. (2019). GFN2-xTB—An
Accurate and Broadly
Parametrized Self-Consistent Tight-Binding Quantum Chemical Method
with Multipole Electrostatics and Density-Dependent Dispersion Contributions. J. Chem. Theory Comput..

[32] Bannwarth C., Caldeweyher E., Ehlert S., Hansen A., Pracht P., Seibert J., Spicher S., Grimme S. (2021). Extended tight-binding
Quantum Chemistry Methods. WIREs Comput. Mol.
Sci..

[33] Lawrence J., Bernal J., Witzgall C. (2019). A Purely Algebraic Justification
of the Kabsch-Umeyama Algorithm. J. Res. Natl.
Inst. Stand. Technol..

[34] Xu X., Truhlar D. G. (2011). Accuracy
of Effective Core Potentials and Basis Sets
for Density Functional Calculations, Including Relativistic Effects,
As Illustrated by Calculations on Arsenic Compounds. J. Chem. Theory Comput..

[35] Lin Y.-S., Li G.-D., Mao S.-P., Chai J.-D. (2013). Long-Range Corrected
Hybrid Density Functionals with Improved Dispersion Corrections. J. Chem. Theory Comput..

[36] Neese F., Wennmohs F., Hansen A., Becker U. (2009). Efficient Approximate
and Parallel Hartree–Fock and Hybrid DFT Calculations. A ‘Chain-of-Spheres’
Algorithm for the Hartree–Fock Exchange. Chem. Phys..

[37] Helmich-Paris B., De Souza B., Neese F., Izsák R. (2021). An Improved
Chain of Spheres for Exchange Algorithm. J.
Chem. Phys..

[38] Casanova-Páez M., Goerigk L. (2020). Assessing the Tamm–Dancoff Approximation, Singlet–Singlet,
and Singlet–Triplet Excitations with the Latest Long-Range
Corrected Double-Hybrid Density Functionals. J. Chem. Phys..

[39] Garcia-Ratés M., Neese F. (2020). Effect of the Solute Cavity on the Solvation Energy and Its Derivatives
within the Framework of the Gaussian Charge Scheme. J. Comput. Chem..

[40] Du J., Ma Y., Ma J., Li S., Li W. (2022). Transition Orbital
Projection Approach for Excited State Tracking. J. Chem. Phys..

[41] Steinmetzer J., Kupfer S., Gräfe S. (2021). Pysisyphus: Exploring Potential Energy
Surfaces in Ground and Excited States. Int.
J. Quantum Chem..

[42] Kasha M. (1950). Characterization
of Electronic Transitions in Complex Molecules. Discuss. Faraday Soc..

[43] Plasser F. (2020). TheoDORE:
A Toolbox for a Detailed and Automated Analysis of Electronic Excited
State Computations. J. Chem. Phys..

[44] Messina F., Sciortino L., Popescu R., Venezia A. M., Sciortino A., Buscarino G., Agnello S., Schneider R., Gerthsen D., Cannas M., Gelardi F. M. (2016). Fluorescent Nitrogen-Rich
Carbon Nanodots with an Unexpected β-C_3_ N_4_ Nanocrystalline Structure. J. Mater. Chem.
C.

[45] Ghosh S., Chizhik A. M., Karedla N., Dekaliuk M. O., Gregor I., Schuhmann H., Seibt M., Bodensiek K., Schaap I. A. T., Schulz O., Demchenko A. P., Enderlein J., Chizhik A. I. (2014). Photoluminescence of Carbon Nanodots:
Dipole Emission Centers and Electron–Phonon Coupling. Nano Lett..

[46] Arcudi F., Đorđević L., Prato M. (2016). Synthesis Separation,
and Characterization of Small and Highly Fluorescent Nitrogen-Doped
Carbon NanoDots. Angew. Chem., Int. Ed..

[47] Nijegorodov N., Mabbs R., Downey W. S. (2001). Evolution of Absorption, Fluorescence,
Laser and Chemical Properties in the Series of Compounds Perylene,
Benzo­(Ghi)­Perylene and Coronene. Spectrochim.
Acta, Part A.

