# Supplementary material for: How Progressive Hydrogenation Shapes the Optical Response of Carbon Clusters
Source: J Phys Chem A. 2026 Oct 7;130(42):8216–30. doi: 10.1021/acs.jpca.6c03043 (PMC13700102; doi:10.1021/acs.jpca.6c03043)
Supplement: Supplementary file 1 [file jp6c03043_si_001.pdf]

How Progressive Hydrogenation Shapes the Optical Response of Carbon Clusters

Francesca D'Ambrosio[1], Alice Frustaci[1], Enrico Bodo[1]*

[1]Chemistry Department, University of Rome "La Sapienza",
P. Aldo Moro 5, 00185, Rome, Italy.

Supporting Information

Table S1. Selected vertical excited states at the ground state geometry of the C_{20} model systems, namely $C_{20}H_5$, $C_{19}H_5N$, and $C_{20}H_7N$. We report the vertical excitation energies, associated wavelength and oscillator strengths. D and S stand for doublet and singlet respectively. The excited states were selected either as the lowest-energy optically active state or as those with the highest oscillator strength.

Cluster	State	Energy (eV)	λ (nm)	f	
**$C_{20}H_5$**	D_3	2.62	474.0	0.0015	lowest
	D_{20}	4.28	298.7	0.0067	bright
	D_{25}	4.49	276.3	0.0592	bright
**$C_{19}H_5N$**	S_1	2.85	435.2	0.0383	lowest
	S_2	3.06	404.8	0.2727	bright
	S_4	3.34	370.8	0.2528	bright
**$C_{20}H_7N$**	S_1	2.53	490.8	0.0613	lowest
	S_4	3.61	343.5	0.1633	bright
	S_7	4.09	303.5	0.2742	bright

Table S2. Results of the excited-state geometric optimizations performed on the selected states of $C_{20}H_5$, $C_{19}H_5N$, and $C_{20}H_7N$ in Table S1. For the state listed in Table S1 for which we found a minimum, the table reports the lowest-energy transition of the same multiplicity with non-zero oscillator strength, evaluated at the geometry obtained by optimization of that state. The corresponding emission energy, wavelength, and oscillator strength are reported.

Cluster	Initial state	Final state	Transition	Energy (eV)	λ (nm)	f
**$C_{20}H_5$**	D_3	D_1	$D_0 \leftarrow D_1$	1.76	704.8	0.0019
	D_{20}	D_{16}	$D_0 \leftarrow D_3$	2.74	452.2	0.0016
**$C_{19}H_5N$**	S_1	S_1	$S_0 \leftarrow S_1$	1.96	634.0	0.0430
	S_2	S_1	$S_0 \leftarrow S_1$	1.96	633.5	0.0430
	S_4	S_1	$S_0 \leftarrow S_1$	1.75	707.3	0.0008
**$C_{20}H_7N$**	S_1	S_1	$S_0 \leftarrow S_1$	1.35	915.8	0.0984

Table S3. Emission positions and corresponding oscillator strengths for $C_{30}H_9$ and $C_{30}H_{11}N$. In both cases optimization of D_1 and S_1 led to emission with very small oscillator strengths and far from the experimental detectability window. Optimization of D_2 and S_2 led to significantly larger oscillator strengths and are therefore identified as more plausible emissive states.

Cluster	Optimized state	Transition	Energy (eV)	λ (nm)	f
**$C_{30}H_9$**	D_1	$D_0 \leftarrow D_1$	0.56	2226.0	0.0015
	D_2 (becomes D_1)	$D_0 \leftarrow D_1$	1.02	1213.0	0.0236
**$C_{30}H_{11}N$**	S_1	$S_0 \leftarrow S_1$	0.11	11244.6	0.0012
	S_2	$S_0 \leftarrow S_2$	1.17	1060.2	0.0521

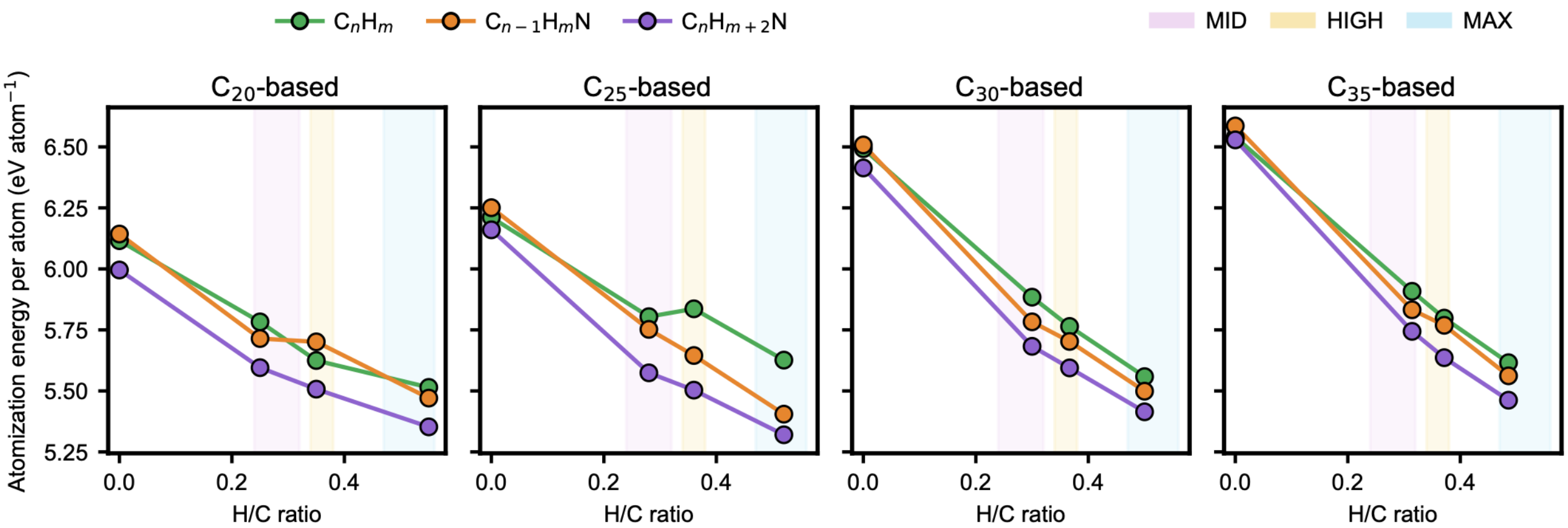

Figure S1. Comparison between unsaturated and partially saturated clusters in terms of atomization energy per atom. The average atomization energy per atom is plotted as a function of the H/C ratio for C_{20}, C_{25}, C_{30} and C_{35} based structures, including here also the unsaturated species (H/C=0). Symbols correspond to the mean values obtained from the five selected isomers for each composition. Colors indicate the cluster type: pristine C_nH_m in green, nitrogen-doped $C_{n-1}H_mN$ in orange and amino-functionalized $C_nH_{m+2}N$ in purple. Shaded areas identify the three ranges of (MID, HIGH, and MAX) used to classify the degree of hydrogenation, expressed as the H/C ratio.

Structural parameters and principal component analysis

Four structural descriptors, gyration radius, asphericity, ring index and largest conjugated-domain size, were computed directly from the optimized geometries file in .xyz using a custom Python workflow based on the ASE and NetworkX libraries. The radius of gyration, r_g, and the asphericity parameter, β, were evaluated from the principal moments of inertia following the Hill–Wheeler formalism, consistent with the definitions adopted by Pla et al. (J. Phys. Chem. A 2021, 125, 5273–5288). Specifically, the inertia tensor was computed with respect to the center of mass, and its eigenvalues $I_1 \geq I_2 \geq I_3$ were used to calculate the radius of gyration:

$$r_g = \sqrt{\frac{I_1 + I_2 + I_3}{2}}$$

The a-sphericity parameter was computed as:

$$\beta = \frac{I_1 - I_3}{I_1 + I_2 + I_3}$$

This definition yields β=0 for perfectly spherical structures and approaches 1 for highly elongated geometries. The largest conjugated-domain descriptor, $N_{\text{conj}}^{\text{max}}$, is defined as the number of carbon atoms in the largest connected component of a graph G_{conj} representing the carbon subnetwork geometrically compatible with π-conjugation:

$$N_{\text{conj}}^{\text{max}} = \max_{C_k \in \mathcal{C}(G_{\text{conj}})} |\ C_k\ |.$$

The graph vertices correspond to carbon atoms with local coordination and geometry compatible with participation in a conjugated network, while edges connect bonded carbon pairs satisfying $d_{CC} \leq 1.50$Å.

We use $N_{\text{conj}}^{\text{max}}$, rather than the total sp^2 fraction as the primary descriptor because the latter quantifies the abundance of locally sp^2-like carbon sites but does not distinguish between a single extended conjugated network and several disconnected sp^2 fragments. $N_{\text{conj}}^{\text{max}}$ instead provides a more direct measure of the spatial extent of the conjugated framework relevant to electronic delocalization and optical absorption.

The ring index $R_{5/6}$ was determined through a graph-based analysis of the carbon framework. A connectivity graph was constructed using interatomic distances and covalent radii, applying a cutoff defined as 1.20 times the sum of the covalent radii of each atom pair. Only carbon atoms were retained to build the corresponding subgraph, and simple cycles were identified using a cycle-basis algorithm. The numbers of five- and six-membered rings (N_5 and N_6, respectively) were extracted, and the structural descriptor was defined as:

$$R_{5/6} = N_5 + N_6$$

This quantity therefore represents the total number of pentagonal and hexagonal rings in the carbon framework and provides a measure of the degree of graphitic ordering within the clusters.

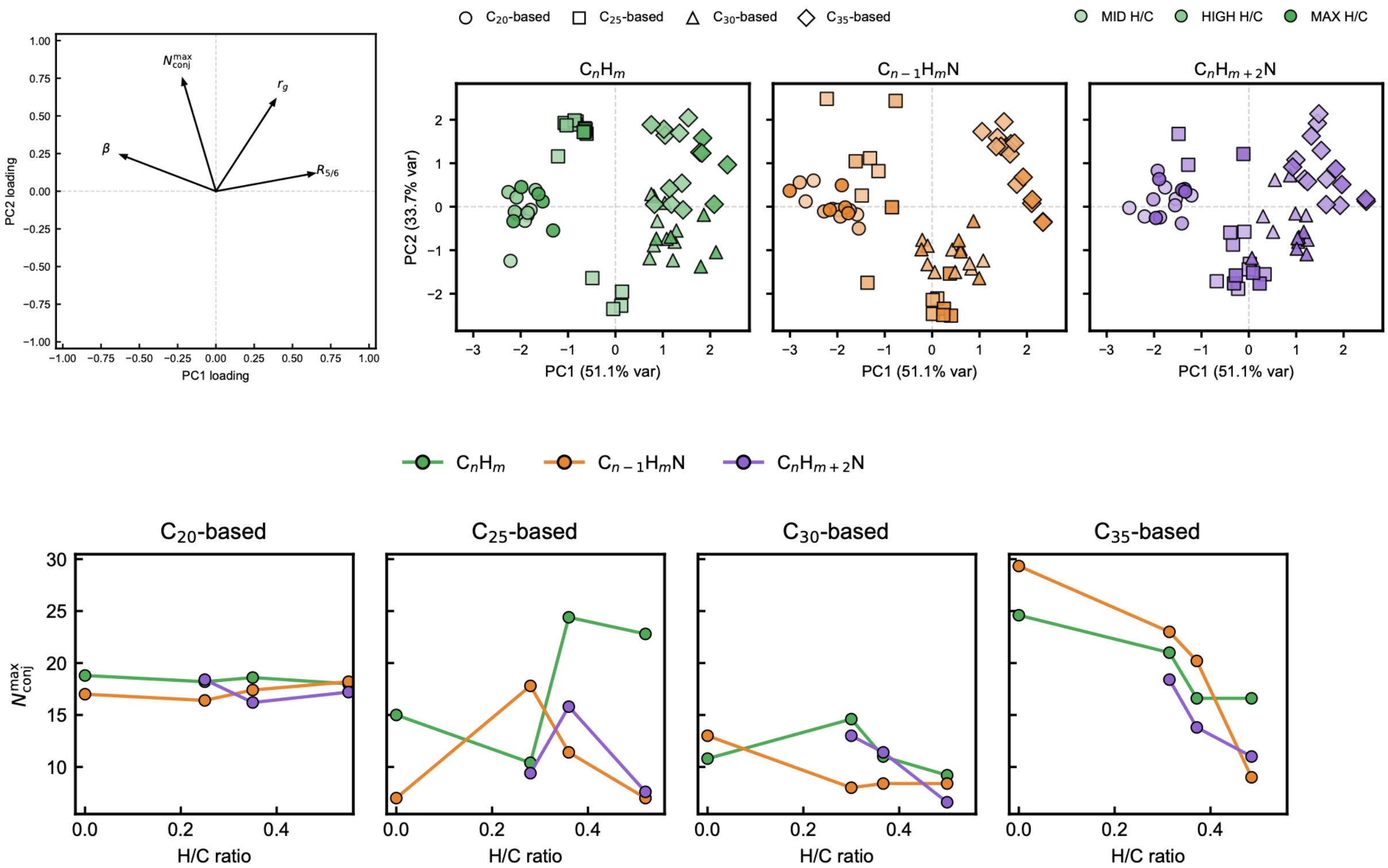

Figure S2. Top Left: loadings of the four structural descriptors in the PC1–PC2 plane: gyration radius (r_g), asphericity parameter (β), fused-ring descriptor ($R_{5/6}$), and largest conjugated-domain size ($N_{\mathrm{conj}}^{\mathrm{max}}$). PC1 is mainly associated with $R_{5/6}$and β, whereas $N_{\mathrm{conj}}^{\mathrm{max}}$contributes most strongly to PC2 together with r_g, indicating that the extent of the surviving conjugated domain provides a structural contribution distinct from the purely geometric descriptors. Top Right, three graphs: principal component analysis of the hydrogenated cluster set shown separately for the three chemical families: pristine C_nH_m, graphitic-N $C_{n-1}H_mN$, and NH_2-functionalized $C_nH_{m+2}N$. Marker shape identifies the cluster size (C_{20}, C_{25}, C_{30}, and C_{35}), while colour intensity indicates the hydrogenation regime (MID, HIGH, and MAX H/C). The projection corresponds to the same revised PCA reported in Figure 4 of the main text, with PC1 and PC2 accounting for 51.1% and 33.7% of the total variance, respectively. Bottom, 4 graphs: evolution of the largest conjugated-domain size, $N_{\mathrm{conj}}^{\mathrm{max}}$, as a function of the H/C ratio for the C_{20}, C_{25}, C_{30}, and C_{35} based systems. Green, orange, and purple curves denote pristine C_nH_m, graphitic-N $C_{n-1}H_mN$, and NH_2-functionalized $C_nH_{m+2}N$ families, respectively. Unsaturated structures (C_n) are included for the pristine and graphitic-N series.

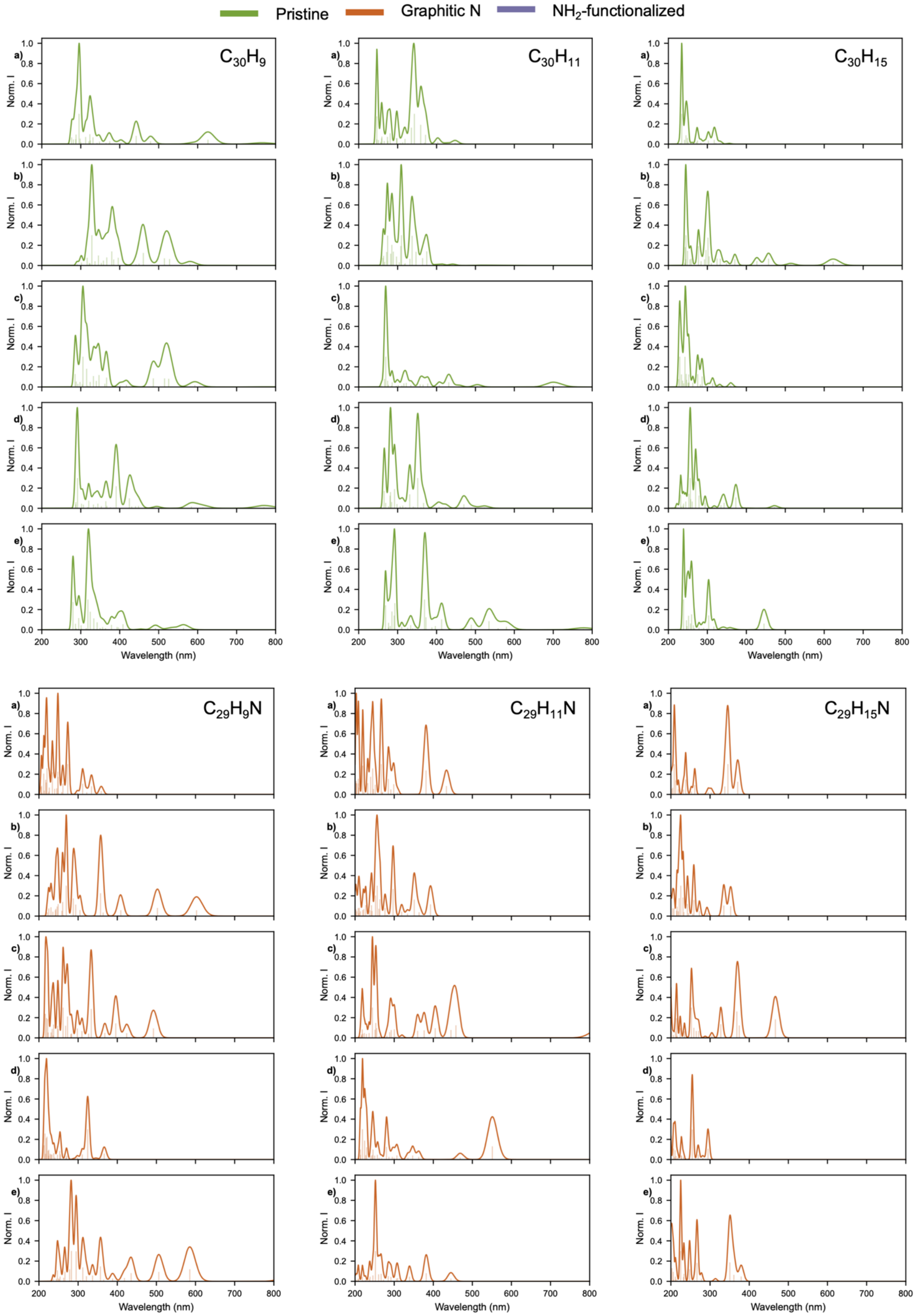

Pristine
Graphitic N
NH$_2$-functionalized
$C_{30}H_9$
$C_{30}H_{11}$
$C_{30}H_{15}$
$C_{29}H_9N$
$C_{29}H_{11}N$
$C_{29}H_{15}N$
a)
b)
c)
d)
e)
Norm. I
Wavelength (nm)

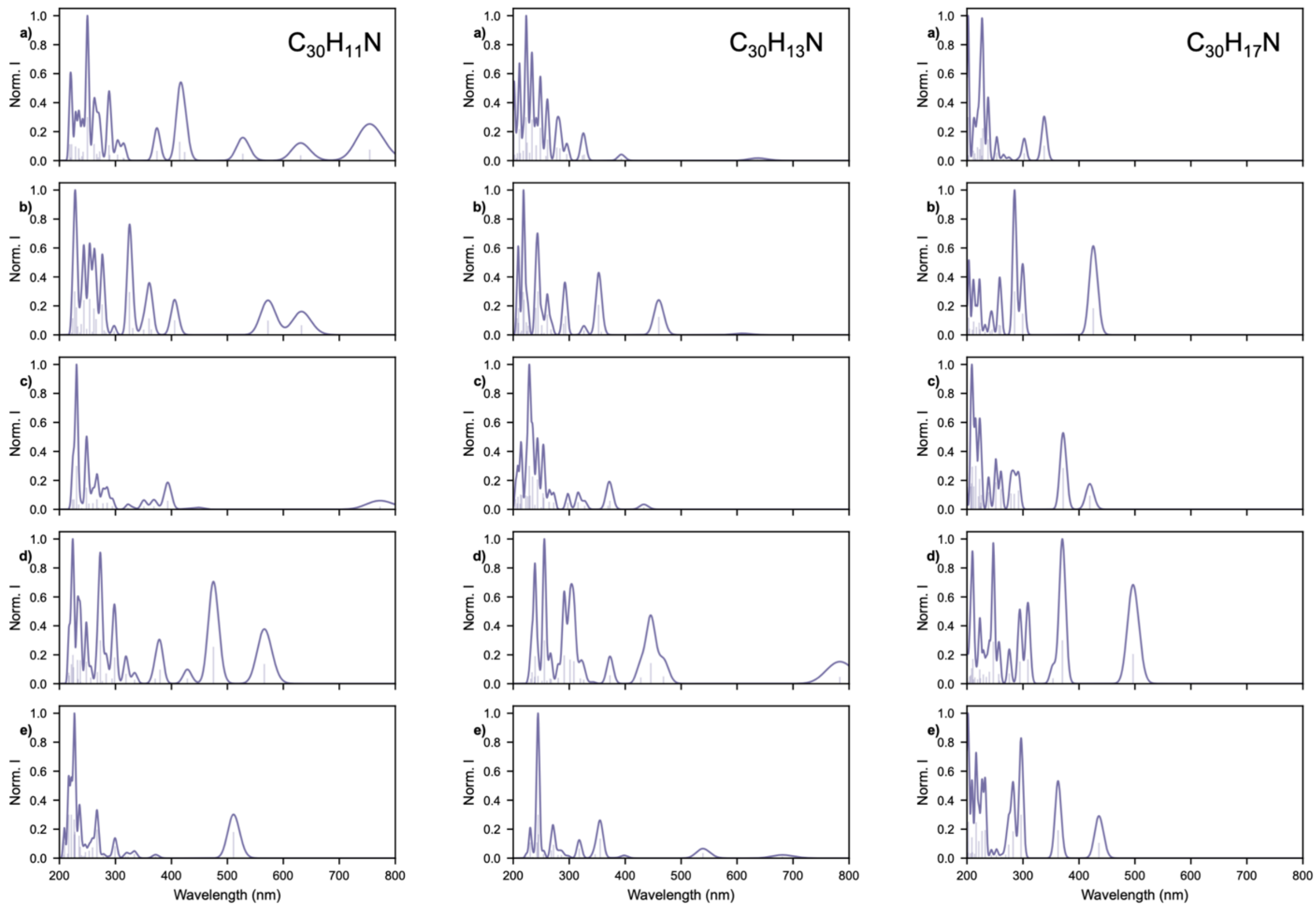

Figure S3. Calculated absorption spectra of the five selected isomers for all the carbon clusters considered. The specific chemical formula for each system is reported in the top panel. For clarity, spectra are drawn in green for the pristine C_nH_m, in orange for the nitrogen-doped $C_{n-1}H_mN$ and in purple for the amino-functionalized $C_nH_{m+2}N$ systems. Vertical sticks correspond to individual TDDFT transitions, while the continuous curves represent spectra obtained by Gaussian broadening (FWHM = 0.12 eV). Each spectrum is normalized arbitrarily to one in order to allow for a comparison between different isomers and chemical compositions.

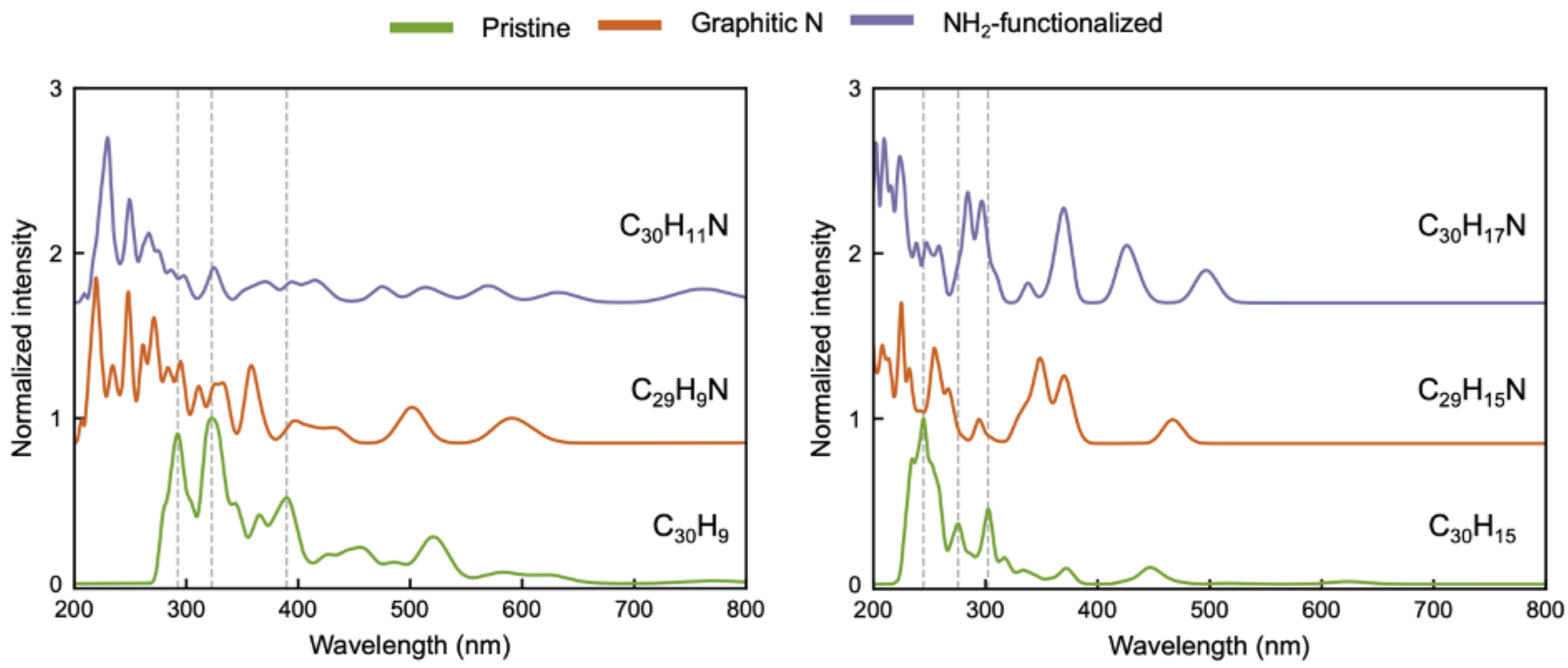

Figure S4. Effect of chemical composition on the absorption spectra of carbon clusters at different hydrogenation levels. The left panel reports the spectra of pristine $C_{30}H_9$, its N-doped and functionalized variants. The right panel reports the spectra of $C_{30}H_{15}$ and its variant. Each spectrum corresponds to the average absorption profile obtained from the five selected isomers. Spectra are normalized to their maximum intensity and vertically shifted for clarity. Dashed vertical lines mark the positions of the three most intense absorption bands of the pristine clusters (green).

Table S5. Natural transition orbital analysis of selected absorption and emission transitions of representative C_{30} based clusters at the indicated minimum geometry. The dominant NTO hole–particle pairs contributions are reported together with their weights. Fragment-based TheoDORE analysis was possible only for the closed-shell structures due to program limitation. PRNTO is the natural-transition-orbital participation ratio and provides a measure of the effective number of NTO hole–particle pairs contributing to a transition; values close to unity indicate that the transition is largely described by a single NTO pair. RMSeh is the root-mean-square electron–hole separation and describes the spatial extent of the excitation; it should not be interpreted as a direct charge-transfer distance. CT is the total interfragmentary charge-transfer contribution obtained from the TheoDORE transition-density analysis and corresponds to the sum of the off-diagonal contributions of the fragment-resolved Ω matrix. The "Main CT component" column reports the largest directional off-diagonal contribution.

System	Geom	Transition	E (eV)	Dominant NTO pair (weight)	PRNTO	RMSeh (Å)	CT (%)	Main CT component (%)	Assignment
**$C_{30}H_9$**	D_0	$D_0 \rightarrow D_2$	1.98	94α→95α (0.77); 93β→94β (0.19)	—	—	—	—	delocalized π→π*
		$D_0 \rightarrow D_5$	2.80	93β→94β (0.51); 94α→95α (0.28)	—	—	—	—	mixed delocalized π→π*
	D_2	$D_0 \leftarrow D_1$	1.05	94α→95α (0.97)	—	—	—	—	delocalized/ local excitation
**$C_{29}H_9N$**	S_0	$S_0 \rightarrow S_1$	3.45	94→95 (0.801); 93→96 (0.147)	1.504	3.117	9.10	N→framework 7.34	local excitation with modest CT
		$S_0 \rightarrow S_2$	3.71	94→95 (0.683); 93→96 (0.278)	1.836	3.071	7.44	N→framework 6.07	local excitation with modest CT
	S_1	$S_0 \leftarrow S_1$	2.77	94→95 (0.95)	1.114	3.219	10.61	N→framework 8.33	local excitation with modest CT
**$C_{30}H_{11}N$**	S_0	$S_0 \rightarrow S_1$	1.64	98→99 (0.973)	1.057	3.456	1.68	NH_2→framework 0.96	local excitation
		$S_0 \rightarrow S_2$	1.96	98→99 (0.901); 97→100 (0.088)	1.219	3.523	1.01	NH_2→framework 0.80	local excitation
	S_2	$S_0 \leftarrow S_2$	1.23	98→99 (0.910) 97→100 (0.077)	1.200	3.514	1.20	NH_2→framework 0.95	local excitation
